# Therapeutic Applications of Nanomedicine: Recent Developments and Future Perspectives

**DOI:** 10.3390/molecules29092073

**Published:** 2024-04-30

**Authors:** Farah Rehan, Mingjie Zhang, Jun Fang, Khaled Greish

**Affiliations:** 1 Department of Molecular Medicine, Al-Jawhara Centre for Molecular Medicine, College of Medicine and Medical Sciences, Arabian Gulf University, Manama 323, Bahrain; farahr@agu.edu.bh; 2 Faculty of Pharmaceutical Sciences, Sojo University, Ikeda 4-22-1, Nishi-ku, Kumamoto 860-0082, Japan; xuanjie1101@gmail.com; 3 Department of General Surgery, Shengjing Hospital of China Medical University, Shenyang 110004, China

**Keywords:** nanomedicine applications, immunotherapy, theranostic, vaccine development, gene therapy, tissue engineering

## Abstract

The concept of nanomedicine has evolved significantly in recent decades, leveraging the unique phenomenon known as the enhanced permeability and retention (EPR) effect. This has facilitated major advancements in targeted drug delivery, imaging, and individualized therapy through the integration of nanotechnology principles into medicine. Numerous nanomedicines have been developed and applied for disease treatment, with a particular focus on cancer therapy. Recently, nanomedicine has been utilized in various advanced fields, including diagnosis, vaccines, immunotherapy, gene delivery, and tissue engineering. Multifunctional nanomedicines facilitate concurrent medication delivery, therapeutic monitoring, and imaging, allowing for immediate responses and personalized treatment plans. This review concerns the major advancement of nanomaterials and their potential applications in the biological and medical fields. Along with this, we also mention the various clinical translations of nanomedicine and the major challenges that nanomedicine is currently facing to overcome the clinical translation barrier.

## 1. Introduction

Physicist Richard Feynman initially introduced the idea of nanotechnology in 1959 during a talk about creating objects at the atomic and molecular levels. Nowadays, scientists believe that nanotechnology is the most promising technological advancement of the twenty-first century. They have studied it as a potential new method for studying medical conditions. Public funding for nanotechnology research and development has risen over the last ten years, indicating that nanotechnology will usher in a new era of productivity and prosperity [1]. Moreover, the application of nanomedicine has opened up previously unexplored potential, especially in cancer treatment; it offers precision targeting, increased efficacy, and decreased adverse effects.

Numerous nanoparticles (NPs), including liposomes, polymeric NPs, and inorganic NPs, are now providing benefits in the therapeutic field, including improved in vitro and in vivo drug stability, therapeutic efficacy, and ease of surface modification [2]. Most recently, biospecific molecules can now be conjugated with NPs through chemical or physical techniques, and NPs can be employed in the utilization of certain biological events, such as the antibody–antigen interaction, the receptor–ligand interaction, and DNA–DNA hybridization [3]. To focus medication delivery on target areas and prevent enzymatic degradation, engineered nanoparticles (NPs) can be made to control drug release and target or avoid specific interactions with different cells [2]. Although many of the aforementioned issues are resolved by nanoparticle-mediated delivery methods, there are a few things to keep in mind when utilizing them, especially regarding nanoparticle design. Particle size, shape, hydrophobicity, and surface charge all have a sensitive impact on how well nanoparticles reach targets. Large nanoparticles (>100 nm) can become retained in the extracellular matrix (ECM), while small nanoparticles (<5 nm) may leak out of blood vessels during circulation. On the other hand, medium-sized nanoparticles, which range from around 5 to 100 nm, remain in the bloodstream and are efficiently transported to target sites. Particle shape (as well as size) is a significant component that affects the drainage of nanoparticles from the lymph nodes. The majority of previous nanoparticle formulations were spherical, but a variety of different forms, including rods, prisms, cubes, stars, and discs, have been produced recently thanks to advancements in nanoparticle engineering. In addition to particle size and shape, the carrier’s surface charge is important for cellular internalization and immune response activation. Surface charge may have an impact on how well cells absorb nanoparticles. Positively charged nanoparticles typically elicit a stronger immunological response compared to their neutral or negatively charged counterparts. However, because they are frequently trapped in the negatively charged ECM, positively charged nanoparticles show decreased tissue permeability [4]. Thus, specifically engineered nanoparticles can provide various benefits over conventional therapeutics, including protecting a therapeutic payload from biological degradation, targeting tumor cells via active targeting, which will minimize off-target normal cell toxicity, and enhancing in vivo stability and bioavailability, and such nanoparticles have been smartly programmed to release their payload at the site of interest [5]. Moreover, the specific application of nanomaterials is determined by their surface chemistry (composition), surface physics (topography and roughness), surface thermodynamics (wettability and free energy), and toxicological effects [3].

In the realm of nanomedicine applications, a primary focus has been on cancer treatment, leveraging the enhanced permeability and retention (EPR) effect [6]. The history, mechanisms, and future implications of the EPR effect have been thoroughly documented in various articles and book chapters [6,7,8,9,10,11,12]. This effect plays a crucial role in the selective accumulation of nanomedicines within tumor tissues, enhancing their effectiveness while minimizing systemic exposure and side effects. In essence, the EPR effect refers to the phenomenon of selective accumulation of macromolecular agents in tumor tissues, facilitated by the unique anatomical and pathophysiological characteristics of tumor blood vessels. This allows for the preferential permeation and retention of macromolecular drugs within solid tumors following systemic administration. The validity of the EPR effect has been established not only in experimental animal models but also in humans, including cases of liver, renal, and metastatic breast cancers [13,14,15]. Recent studies examining human renal tumors and metastatic breast cancers have demonstrated a significant EPR effect in more than 87% of samples, highlighting the pivotal role of this effect in the advancement of anti-cancer nanomedicines [13,14]. Based on the EPR effect, nanomedicines exhibit many beneficial features for targeted cancer therapy, in contrast to those of conventional small molecular anti-cancer drugs, which tend to spread indiscriminately in normal tissues and organs, thus leading to systemic adverse effects [7,8,10]. Nanomedicines, therefore, offer promise in terms of improved treatment options. In the 1990s, the first polymer-conjugated nanomedicine, SMANCS (styrene maleic acid polymer-conjugated neocarzinostatin), was approved in Japan, marking a significant milestone in the development of anti-cancer nanomedicine [15]. Over the past two decades, the field of anti-cancer nanomedicine has experienced substantial growth and advancement [16,17,18]. Many nanomedicines are currently being utilized in clinical settings, with numerous others undergoing clinical trials (see Table 1).

**Figure 1:**
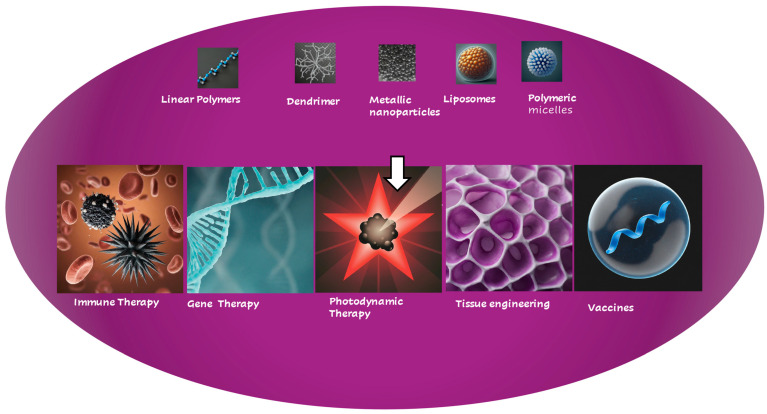
Novel applications of nanomedicine in various biomedical and therapeutic fields.

In recent years, numerous nanomedicines have been designed and developed for advanced, less-invasive cancer therapy. Examples include nanoprobe-based photodynamic therapy [12,19] and boron neutron capture therapy (BNCT) [20,21], as well as magnetic hyperthermia therapy using magnetite [12,19], all of which demonstrate promising therapeutic potential. Additionally, the application of nanomedicine in cutting-edge biomedical fields such as immunotherapy, gene therapy, and preventive medicine has garnered significant attention. A notable example is the success of mRNA vaccines for COVID-19, which utilize lipid nanoparticles as the delivery system [22]. In this context, this review explores the promising applications of nanomedicine in advanced cancer therapy and other biomedical fields, as depicted in Figure 1.

Through an explanation of the fundamental ideas, current developments, and potential future directions, we highlighted the impact of nanomedicine through their advanced properties, leading us to new paradigms in precision healthcare and better patient outcomes.

### 1.1. Nanomedicine: Applications in Photodynamic Therapy

#### 1.1.1. Nano-Designed Photosensitizers, a Promising Therapeutic Approach for Cancer

Despite the continuous expansion of tumor treatment methods, significant challenges persist, including non-specificity, drug resistance, low response rates, and severe toxicity-related side effects [23]. Photodynamic therapy (PDT) offers a non-invasive and targeted alternative, whereby light-activated photosensitizers (PS) generate reactive oxygen species (ROS), leading to tumor cell death. PDT holds promise as an effective approach to address the shortcomings of traditional tumor treatment strategies [24]. However, the clinical translation of PDT is hampered by the limitations of traditional photosensitizers mostly due to the lack of tumor selectivity, resulting in its relatively limited application in current clinical practice [25]. In this regard, the emergence of the nanophotosensitizer (NanoPS) serves as a means of overcoming these challenges by taking advantage of the enhanced permeation and retention (EPR) effect to target tumor, offering significant potential to enhance PDT efficacy and specificity [19]. Moreover, because most PSs exhibit fluorescent nature, it is possible to differentiate tumor and normal tissues via fluorescent imaging, i.e., photodynamic diagnosis (PDD).

#### 1.1.2. NanoPS for Tumor Targeting

Compared to traditional PS, NanoPSs exhibit superior tumor-targeting capabilities due to their nanoscale size and surface modifications [26]. NanoPSs can further be functionalized with specific targeting ligands or antibodies on their surface, enabling specific recognition and binding to tumor cells, thus enhancing therapeutic efficacy and minimizing damage to normal tissues [26]. Through strategies such as EPR-effect-mediated passive accumulation and active targeting using ligands or antibodies, NanoPSs can precisely localize within tumors, minimizing off-target effects and enhancing therapeutic outcomes [27]. Additionally, the optical properties of NanoPSs can be tuned by controlling their morphology, size, and structure, allowing for maximal absorption of photosensitizers at specific wavelengths, thereby improving PDT efficiency and achieving more precise tumor treatment [28].

#### 1.1.3. Clinical Applications of PDT and the Potential of NanoPSs

In the past two decades, clinical research related to PDT has experienced rapid development, with several PS now approved for clinical treatment. For instance, Methyl aminolevulinate (MAL) [29] and 5-ALA [30] have been approved by the Food and Drug Administration (FDA) for treating actinic keratosis, basal cell carcinoma (BCC), superficial and nodular basal cell carcinoma, and squamous cell carcinoma (SCC), respectively. Radachlorin^®^ has also obtained approval from the Ministry of Health of the Russian Federation (MHRF) for skin cancer treatment [31]. Temoporfin has been approved by the European Medicines Agency (EMA) for palliative treatment of advanced head and neck squamous cell carcinoma [32]. PDT has a relatively mature application foundation in the treatment of cutaneous malignancies, particularly for non-melanoma skin cancers like basal cell carcinoma and squamous cell carcinoma [33]. Its localized treatment approach not only yields favorable cosmetic outcomes, reducing the impact on patients’ appearance, but also demonstrates good anti-tumor effects and lower recurrence rates [34]. In the realm of thoracic malignancies, PDT, as one of the essential therapeutic modalities, has been approved and widely utilized, particularly for early-stage non-small-cell lung cancer [35]. In various other domains, such as head and neck malignancies, urogenital malignancies, breast malignancies [36], intracranial malignancies [37], and gastrointestinal malignancies, PDT has shown extensive potential. With the rational design of novel nanoscale photosensitizers, such as overcoming the challenges of the blood–brain challenges using ultra-small NanoPSs, the prospects for PDT in brain tumor treatment are increasingly promising [38]. Additionally, clinical research on PDT for various malignancies, including ovarian cancer, endometrial cancer, cervical cancer, osteosarcoma, and hematologic malignancies, is vigorously ongoing. With advancing research, PDT presents promising prospects in an expanding array of cancer treatments. In summary, PDT, as a localized treatment modality, coupled with the development of novel photosensitizers and nanotechnology, holds the potential to bring more personalized and precise options for treating various malignancies, further improving treatment outcomes, and enhancing patient survival rates and quality of life.

#### 1.1.4. Versatile Therapeutic Strategies Utilizing NanoPSs

The continuous development of NanoPSs has endowed certain types with not only photosensitivity but also sonosensitivity and photothermal properties [39,40]. These characteristics enable PDT to be combined with sonodynamic therapy (SDT) or photothermal therapy (PTT), providing sufficient flexibility to integrate with other treatment modalities to achieve synergistic effects [41,42]. For example, research by Liao et al. has found that Chlorin e6 exhibits efficient photosensitivity and sonosensitivity, playing a crucial role in sonodynamic therapy combined with PDT [43]. Additionally, utilizing NanoPSs for the co-delivery of chemotherapy drugs, immune modulators, or gene therapy agents enables the realization of multimodal cancer treatment strategies [44]. Furthermore, integrating imaging probes into NanoPSs allows for real-time monitoring of treatment responses, facilitating personalized treatment approaches [45]. Table 2 summarize the therapeutic strategies and challenges using NanoPSs.

#### 1.1.5. Challenges and Future Perspective

The extensive application of NanoPSs in cancer therapy has made significant strides, demonstrating outstanding efficacy not only in basic research but also in clinical studies. The therapeutic efficacy of cancer treatment is closely associated with various factors such as the characteristics of NanoPSs themselves, their transport processes in the organism, and cellular target damage. These factors include the loading and functional modification of small drug molecules, the in vivo transport and tumor accumulation of nanomedicines, the intratumoral penetration and microenvironmental control release, and the specific damage to tumor cells and the biosafety of normal tissues [68]. However, despite significant progress, the widespread clinical application of NanoPS-mediated PDT still faces several challenges. These challenges include concerns about biocompatibility, long-term safety, standardization of synthesis methods, and regulatory approval processes. Future research directions may focus on developing multifunctional NanoPS platforms, refining targeting strategies and optimizing treatment regimens, investigating the mechanisms of drug transport and action at various stages of treatment in vivo, to maximize therapeutic efficacy while minimizing adverse reactions.

In general, currently, there are relatively few NanoPSs approved for clinical use, with most still in the stage of basic research or clinical trials. As research progresses, an increasing number of clinical experiments have been initiated. It can be foreseen that with the rapid development of basic research on NanoPSs in PDT anti-tumor therapy, there will be more related clinical trials in the future, striving to apply more NanoPSs to clinical practice.

### 1.2. Nanomedicine: Applications in Immunotherapy

Immunotherapy started in the 19th century when it was observed that some patients with sarcomas had tumor regression following a streptococcus pyogenes infection. This led to the use of Coley’s toxin to treat some sarcoma patients with occasional complete responses in some patients [69]. The goal of immunotherapy is to utilize the host’s immune response to achieve a long-lasting therapeutic effect. However, it is still difficult to manage the severe side effects of immunotherapeutics, which might induce autoimmune and non-specific inflammation [70].

NPs, both synthetic and naturally produced, have become the subject of a great deal of research in the field of immunotherapy in recent decades because of their unique physical and chemical characteristics [71]. By using nanomedicines and biomaterials, it is becoming possible to deliver immunomodulatory agents to the desired location in a targeted manner, with many advantages such as improved pharmacokinetics, increased therapeutic efficacy, and minimized dose-dependent systemic toxicity [5,70].

To maximize the effectiveness of immunotherapeutic drugs, NPs should be properly engineered to target regions of interest preferentially from the site of administration (parenteral or mucosal vaccination routes are frequent routes of administration). Various strategies mentioned below are being employed to enhance the immunogenic effects of nanomedicine.

#### 1.2.1. Targeting the Tumor Immune Microenvironment

The use of nanomedicines that alter the tumor immune microenvironment (TIME) is a crucial tactic for boosting the effectiveness of anti-cancer immunotherapy [72]. Hypoxia is caused by the rapidly expanding tumor cells and distorted blood vessels in the TIME. This leads to the accumulation of immunosuppressive cells, such as regulatory T cells (Tregs) and myeloid-derived suppressor cells (MDSCs), as well as the secretion of immunosuppressive factors like transforming growth factor β (TGF-β) and vascular endothelial growth factor (VEGF), which recently showed to suppress dendritic cell maturation and t cell infiltration. By focusing on the main elements of the TIME, nanoparticles may alter the immunosuppressive milieu surrounding cancer cells. Furthermore, enhancing the infiltration, proliferation, maturation, survival, and/or activation of effector immune cells such as cytotoxic T cells, boosts the effectiveness of immunotherapy by blocking immunosuppression in the TIME [72,73]. Chen et al. [74]. used albumin-coated MnO_2_ to create pH/H_2_O_2_ dual-responsive nanoparticles. Oxygen was produced by MnO_2_ reacting with H_2_O_2_ and H^+^ when it penetrated the tumor. By reducing the hypoxic state, they improved the efficaciousness of photodynamic treatment and chemotherapy.

Furthermore, major players in the TIME, tumor-associated macrophages (TAMs) initiate anti-tumor immunity during the tumor initiation stage; however, once the tumor is established, they play a role in tumor angiogenesis, immune suppression, invasion, and metastasis. This disparity may arise from the malleability of macrophages, which cause TAMs to move from M1 to M2 polarized states [73,75]. Leonard et al. used liposomes loaded with the CRISPR complex to silence the mammalian target of the rapamycin (mTOR) pathway, an essential cellular signaling pathway involved in many important physiological functions, such as cell growth, proliferation, and autophagy. This caused the macrophages to polarize from the M2 phenotype to the M1 phenotype and hence improve the therapeutic effect of chemotherapy [70].

#### 1.2.2. Targeting and Reprogramming T-Cells

T cells are essential to the adaptive immune response and are part of the lymphocytes. Immune-mediated cell death is one of the T cells’ roles. T cells can be divided into two main subtypes: CD8+ and CD4+ T cells. Cytotoxic T cells, commonly referred to as CD8+ T cells, specifically destroy cancerous and virus-infected cells. Conversely, CD4+ T cells influence other immune cells through cytokines; or, by deciding whether and how the immune system reacts to a perceived threat, they indirectly contribute to the death of infected cells. Because T cells contain multiple cell markers on their surface and are involved in the immune response to a variety of diseases, including cancer, there are multiple target methods that can be used to treat different diseases [76]. The use of RNA interference (RNAi) to down-regulate particular genes and modify T cell activity has great promise for developing tailored therapeutics for a variety of immune-related conditions, such as cancer, inflammation, autoimmune, and viral infections. Ramishetti et al. describe a unique approach that uses targeted lipid NPs (tLNPs) to precisely deliver siRNAs to murine CD4+ T cells. Anti-CD4 monoclonal antibody was used to surface-functionalize the tLNPs, allowing the siRNAs to be delivered to CD4+ T cells only. tLNPs showed selectivity in vivo by only focusing on primary CD4+ T cells and ignoring other cell types. These tLNPs have been synthesized with several lipids to enhance the stability and efficacy of siRNA delivery, hence increasing its efficacy [77]. Moreover, activated T cells (ATCs) express more transferrin receptors, suggesting that ATCs can be targeted with this technique. So, Xie et al. created a polyplex comprising transferrin–polyethylenimine (PEI) and siRNA as a targeted delivery system in order to mute genes linked to inflammation ATCs. They also verified the polyplex’s potential for asthma treatment [76].

The labor-intensive process of reprogramming the immune cells raises the cost of CAR-T therapy to USD 373,000 for Yescarta and USD 475,000 for Kymriah. Nevertheless, nanotechnology provides affordable nanocarriers that can precisely and quickly program tumor-recognizing capabilities into the host T cells, hence resolving these problems. They are used in conjunction with ligands that target lymphocytes to deliver tumor-specific CAR cargo into T cells. Following ingestion, these NPs instruct effector cells in sufficient amounts to induce tumor regression with anti-cancer immunological properties resembling those of the existing CAR-T techniques, which generally transduce cultured T cells ex vivo [78].

#### 1.2.3. Activating and Enhancing NK Cells

The innate immune system’s initial line of defense is made up of natural killer (NK) cells, which are crucial for identifying cancerous cells and defending against viral infections. NK cells make up anywhere from 5% to 20% of all lymphocytes in peripheral blood in people [79]. Although NK-cell-based immunotherapy is very promising, it still encounters significant obstacles such as limited NK cell proliferation and short in vivo lifespan, as well as expensive treatment costs and complicated delivery systems [80]. However, NP-based therapy tries to overcome these obstacles by selectively activating NK cells. According to Chandrasekaran et al. [81], mice’s subcutaneous tumor metastasis was inhibited by TRAIL-decorated liposomes coupled to NK cells within the tumor-draining lymph nodes (TDLN). Additionally, they discovered that NK cells functionalized with TRAIL extended their retention duration in TDLN to cause tumor cells to undergo apoptosis. Later, Gao et al. [82] created polymeric NPs with diselenide-modified RGD peptides that target tumors, allowing for systemic injection for tumor accumulation and radiation exposure for NK-cell-mediated cancer immunotherapy. Carbon nanotubes (CNTs) may also have immunosuppressive effects based on the mode of administration. In C57BL/6 adult mice, the inhalation of CNTs resulted in decreased T cell proliferation, decreased NK cell activity, and increased gene production of IL-10 in the spleen, an anti-inflammatory cytokine. This caused systemic immunosuppression in the mice. Additionally, gold nanorods were administered intranasally to reduce TNF-α, GM-CSF, IL-17, and IL-12p70 and raise IL-9 in contrast to mice infected with the respiratory syncytial virus [83]. In another study, bispecific antibodies (SS-Fc, anti-CD16, and anti-CEA) were used to coat the Ruthenium NPs, which stimulated NK cells to cause necrosis and apoptosis, which, in turn, further activated immunological responses [84].

#### 1.2.4. Activating NKG2D Receptors

NKG2D is an activating receptor that resembles C-type lectin and is widely expressed by CD8+ T, γ δ T, NK, and NKT cells. Mouse murine UL-16 binding protein (ULBP)-like transcript 1, H60, and retinoic-acid-early-induced transcript-1 (RAE-1) are the NKG2D ligands. Since most normal tissues lack NKG2D ligands, which are forced to express by virus-infected or tumor cells, these ligands function as tumor-targeted antigens in immunotherapy [85]. TGF-β inhibitor and selenocysteine nanoemulsion enhance the lytic potential of NK cells by sensitizing NKG2D ligands. The TGF-β inhibitor was successful in blocking the TGF-β/TGF-β RI/Smad2/3 signaling pathway, which led to an increase in the amount of NKG2D ligands on the surface of tumor cells. In γδ T lymphocytes, selenium cysteine inhibits PD-1 expression while promoting the production of NKG2D receptors [84]. Another study demonstrated that NKG2D/NPs coated with mouse and human fragment crystallizable (Fc)-fusion NKG2D (Fc-NKG2D) could, through dose-dependent magnetic cell sorting, target a variety of NKG2D-ligand-positive tumor types in vitro. Multiple tumor types can be targeted with NKG2D/NPs, and the proof-of-concept stage of tumor-targeting iron oxide NPs (IONP) research can be aided by magnetic separation platforms [86].

#### 1.2.5. Targeting Antigen-Presenting Cells

Macrophages, dendritic cells (DCs), and B lymphocytes are examples of antigen-presenting cells (APCs), which are an integral component of the innate immune system and are crucial for both starting and controlling the adaptive response. Its primary duty is to identify, seize, and digest antigens. Over the past 20 years, antigen targeting tactics have been extensively investigated as preventive and therapeutic measures for infectious illnesses, autoimmunity, and cancer. These strategies have been created to improve vaccine efficiency through this mechanism of antigen capture [87]. Antigen presentation on the surface of NPs resembles the natural manner in which viruses present antigen and by managing and delaying the antigen’s release, NPs can regulate the immune system’s exposure to it. Additionally, concurrent loading of the antigen and an adjuvant can improve most proteins’ restricted immunogenicity through NPs. Polymeric NPs, in this regard, have garnered attention among the various nanocarriers that have been introduced for loading and antigen delivery. Various types of polymers, such as poly (esters), poly (α-hydroxy acids), proteins, and polysaccharides, have been examined. Gelatin, which has minimal immunogenicity, is also a good candidate as the vaccine nanocarrier. In this regard, antigens like ovalbumin (OVA) and tetanus toxoid had been immobilized on the surface of gelatin NPs (GNPs), by which the immune response to the antigen can be manipulated by altering the method of antigen loading and the NPs’ surface characteristics [88]. Additionally, PEG is typically added to NPs to reduce their interactions with the reticuloendothelial system [89].

#### 1.2.6. As a Carrier for Immune Checkpoint Inhibitors

The surface proteins on immune cells known as immunological checkpoints function as negative regulators of the immune system’s activation by a variety of antigens. Immune checkpoint molecules are widely expressed on tumor cells as well as immune cells [5,90]. However, the lack of an ideal approach for immunotherapy (IMT), immune-related adverse events (irAEs), and low response rates have restricted research into combination therapies using innovative immune checkpoint inhibitors (ICIs). NPs have become effective instruments for fostering interdisciplinary collaboration. Targeted delivery of ICIs via NPs is both feasible and effective; it removes the main obstacle, enhances treatment efficacy, and justifies further clinical research [90]. Lipid-based NPs (liposomes and LNPs) have been developed more and more in clinical trials for the delivery of RNA and immunotherapeutic drugs, but their use for ICIs has not kept up with the demand [90].

#### 1.2.7. Clinical Development of the Nanoimmunotherapy

Based on the advances made thus far, nanoimmunotherapy has shown promise in treating various diseases. The majority of these results are still in the preclinical stage, with some having received FDA approval and are currently under clinical trials, as indicated in Table 3. Liposomes, polymer NPs, and PEG-drug conjugates are currently approved for the treatment of various immunological disorders [91].

Phase-1/2/3 clinical trials have assessed the safety and efficacy of ALT-803, the recombinant IL-15 superagonist complex nanogel that targets NK and T cells, for treating patients with leukemia or advanced solid tumors (e.g., NSCLC, melanoma, renal carcinoma, colon cancer, and breast cancer). LNP-mRNA technology, whose lipoplex permits the targeted delivery of therapeutic mRNA cancer vaccines to APCs in lymph organs, is the preferred nanoformulation that is presently undergoing clinical studies [92]. Nanobiotix created Hensify^®^ to boost the immune system locally and physically kill malignancies. Nanobiotix is conducting several clinical trials and has been approved by the US FDA to begin a combination trial using PD-1 and NBTXR3 antibodies to treat lung cancer [5]. As of 2023, anti-PD-1/PD-L1 and radiation therapy, along with NBTXR3, are under phase-1 and phase-2 clinical trials to aid in the management of patients with advanced solid malignancies (NCT05039632)). In addition, a clinical trial of a novel RNA-nanoparticle vaccine for the treatment of early melanoma recurrence following adjuvant anti-PD-1 antibody therapy is going on to assess the safety and viability of a tumor-specific RNA-NP vaccination in individuals with stage IIB-IV melanoma who have progressed while receiving adjuvant anti-PD1 (a-PD1) therapy (NCT05264974).

Phase III clinical trials are evaluating tecemotide, an MUC1-specific cancer immunotherapy, for the treatment of stage IIIA/IIIB NSCLC. A dendritic-targeted liposomal vaccination called Lipovaxin-MM also started a phase-1 trial for malignant melanoma. Based on cyclodextrin polymeric nanoparticle (CDP) technology, CRLX101 is a first-in-class nanopharmaceutical with the potential to convert therapy into positive clinical results [93].

**Table 3:** Comprehensive information on nanoparticles currently under clinical trials for immunotherapy.

Product Name	Nanoparticles	Targeting Cells/Disease	Mechanism of Action	Status	References
NBTXR3 in combination with immunotherapy (Anti-PD-1/L-1)	Hafnium oxide crystalline NPs	Solid tumors	To boost the immune system locally and physically kill malignancies	Phase I and II	NCT05039632 [93]
Lipovaxin-MM	Liposomal vaccine	Metastatic melanoma	A new anti-cancer vaccine; is safe and effective in improving the body’s ability to destroy cancer cells in patients	Phase I	NCT01052142 [93]
PLD with IPI-549 and Etrumadenant	Liposomal NPs	Metastatic triple-negative breast cancer (TNBC) or ovarian cancer	Adenosine receptor antagonist that can treat malignancies by preventing adenosine-mediated immunosuppression	Phase I	NCT03719326 [94]
MEPACT/Mifamurtide	Liposomal muramyl tripeptide phosphatidylethanolamine (MTP-PE)	Osteosarcoma	Activates macrophages and monocytes in the tumor microenvironment to modulate innate immunity	Expanded access	NCT04571229 [95]
RALA+PLA-bP (NAS-co-NVP)	PLA NPs	HIV	Induces highly cytotoxic T cells	FDA approved	[96]
JVRS-100	Liposomal DNA complex	Potential adjuvant for influenza vaccines	Improves cross-protection and immunogenicity against fatal viral diseases	Phase 1 completed	NCT00662272 [93]
CRLX101	Cyclodextrin-based polymer	Advanced solid tumors	Suppresses the expression of vascular endothelial growth factor, CD31, and carbonic anhydrase IX in tumor sections, which prevented hypoxia and angiogenesis	Phase I and II completed	NCT00333502 [97]
DOTAP liposome vaccine	Cationic liposomes	Potential vaccine against infectious disease and tumors	Causes immune responses via an antigen-specific Th2 reaction	Phase I	NCT05264974 [98]
RNA-LP	Lipid NPs	Melanoma	Activates inherent pathways to activate APCs and suppress myeloid derived suppressor cells (MDSCs)	Phase I	NCT05264974 [99]
AZD4635	Polymeric nanoparticle	Advanced solid malignancies	Enhances anti-tumor activity by rescuing T cell function	Phase 1 completed	NCT02740985 [100]

#### 1.2.8. Challenges and Perspective

Despite the increased interest in NP-based immunotherapy, a significant problem remains with the clinical translation of these immunostimulatory NPs. When assessing the toxicity of NPs, it is important to consider the fact that some of them can potentially change the intracellular signaling route. When NPs interact with serum proteins, the immune system may identify them as foreign materials and develop an autoimmune response against them. As a result, in order for NPs to be used successfully in clinical settings, they must be designed to be able to prevent the production of ROS, hypersensitivity, or allergic sensitization, and to be easily cleared from the body [101]. The main barrier to adoption is an inadequate comprehension of the nanoparticles’ mode of action with biomolecules. This led to the withdrawal of several nanoformulations from the market, even after receiving FDA approval; some examples of these include Feridex I.V. (Endorem, Ferumoxides), Lumirem (Gastromark), Resovist (Cliavist), Sinerem (Combidex), and Clariscan (PEG-Fero, Feruglose NC100150) [93].

### 1.3. Nanomedicine: Applications in Gene Delivery

Since DNA was discovered to be the fundamental building block of heredity, medicine has sought to modify specific regions of the human genome [102]. The ability to fix mutated genes or site-specific alterations to achieve therapeutic treatment is known as gene therapy, by which a patient’s genome can bee partially altered through the replacement, insertion, or deletion of genetic material [103]. The first approved gene therapy procedure was carried out on September 14 1990 by W. French Anderson and his colleagues at the National Institute of Health (NIH) on a four-year-old girl who was born with severe combined immunodeficiency (SCID) [104]. To date, the US Food and Drug Administration (FDA) has authorized four gene treatments for commercialization in the US: in 2017, voretigene neparvovec (marketed as Luxterna^®^) and onasemnogene abeparvovec-xioi (marketed as Zolgensma^®^) were approved, brexucabtagene autoleucel (marketed as Tecartus^®^) was approved in 2020, and in 2022, etranacogene dezaparvovec (marketed as Hemgenix^®^) was licensed [103].

Since viruses are designed to insert their own genetic information into host cells; thus, they make sense as the most-often-utilized gene-delivery vehicle. However, gene therapy using viruses may induce severe clinical adverse effects, such as the death of a high school student participating in a gene therapy trial at the University of Pennsylvania in 1999 [105]. In this context, researchers are working to create totally synthetic non-viral carriers. Moreover, when compared to viral vectors, non-viral carriers—especially NPs—have shown enormous promise for the targeted delivery of genetic material in the treatment of pancreatic cancer, hereditary transthyretin amyloidosis, and other diseases. Nanocarriers and the target tissue were brought to light by the effective delivery of nucleic acids [106,107]. Since then, NPs have become one of the most exciting developments in biomedical research as a carrier for gene therapy because of their low immunogenicity and toxicity, the simplicity of their production, their larger loading capacities, the lack of unexpected gene integration, and their functionalization with various moieties. By using functionalized NPs, some of the restrictions on the transfection effectiveness of naked plasmid DNA (pDNA) or siRNAs can also be overcome [108]. Currently, biocompatible and more efficient transfection systems are being developed to introduce therapeutic nucleic acids (TNAs) into cells and tissues, such as plasmid DNA or anti-sense oligonucleotides (ASO), or RNA into cells, such as microRNA (miRNA), short hairpin RNA (shRNA), or small interfering RNA (siRNA) [107]. Nevertheless, the US Food and Drug Administration (FDA) has not yet approved any gene treatments based on NPs. Concerns about biodegradation and biocompatibility, aggregation in physiological fluids, non-specific adsorption by non-desired tissues, less effective extravasation to reach target tissues, cellular internalization, and endosomal escape still exist for the clinical application of nanoparticle-based gene therapy [109]. Various NPs employed for gene therapy are discussed below and also mentioned in Table 4.

#### 1.3.1. Inorganic NPs

Recently, strong and adaptable nanocarriers for effective gene delivery applications have been demonstrated by inorganic nanomaterials. Furthermore, inorganic nanomaterials have an appealing array of useful applications, such as facile functionalization with thiol or silane groups to improve interaction with biomolecules through thermal and chemical stability. It is also scalable in synthesis. Three methods are generally used to modify inorganic NPs for the delivery of genes: (i) using positively charged inorganic NPs to form a complex with negatively charged genetic material; (ii) directly conjugating the genetic material on the inorganic NPs with a reactive linker; and (iii) using a cationic amphiphilic polymer derived from the NPs to induce the complexation of the inorganic NPs and the genetic material [106]. Important types of inorganic nanomaterials being employed for the delivery of gene therapy include iron oxide NPs (IONP), superparamagnetic iron oxide NPs (SPION), and gold NPs (AuNPs). In the case of IONP, the particles are drawn to an external magnetic field, which changes the distribution of the particles within the organism [108]. When NPs are delivered through cell compartments using a magnetic field, the efficiency of DNA transfer is increased. Because of this, magnetic iron NPs containing DNA were used in mitochondrial therapies to engage with the mitochondrial translocation protein and cause cell death [108,110]. The development of iron NPs loaded with siRNA for silencing gene therapy is possible because of their small size and variable functionalization, which results in a net positive surface charge that amplifies the impact of siRNA. Fe_3_O_4_-synthesised NPs were recently employed to target B-cell lymphoma-2 (BCL2) in Ca9-22 oral cancer cells, and the gene silencing effect was amplified when combined with magnetotherapy [108,111]. The high payload (owing to large specific surface area), low toxicity, accelerated uptake, rapid endosomal escape, increased half-life, and effective and selective gene silencing of AuNPs have led to their increasing use in both in vitro and in vivo gene therapy applications. RNA aptamers specific to the β-catenin gene, for example, were delivered into the nucleus of cancer cells by Ryou and colleagues [112] using AuNPs. This method effectively induced apoptosis by suppressing the transcriptional activity of β-catenin in the lung cancer cells’ nucleus. AuNPs have also been employed as delivery vectors for siRNA, which engage with their target very specifically and promote a silencing complex without requiring genome integration to function.

Moreover, some forms of functionalization, such as cationic lipid bilayer or cationic quaternary ammonium, can improve SiRNA delivery [113]. The delivery of vectors using carbon nanotubes (CNTs) has shown promise because of their high aspect ratio and ability to cross plasma membranes. Furthermore, the nanoneedle characteristics facilitate their diffusion into the cytosol and shield the gene delivery from enzymatic degradation [106]. In order to enhance lipophilicity for improved internalization, Taghavi et al. [114] synthesized single-walled CNTs loaded with PEG and polyethylenimine (PEI) modified by alkylcarboxylation. The results showed that the nanocarrier could enhance the gene transport of sh-RNA to MCF7 cells. Additionally, the silanol group on the surface of silica-based NPs provides a positive charge for functionalization with nucleic acids, diverting some attention away from viral and non-viral vectors. Utilizing in vitro cellular models, silica NPs were applied in gene delivery systems to enhance the sedimentation of NPs and improve the incorporation of genetic material. The properties of silica as a nanocarrier were enhanced when it was combined with lipids, polymers, or inorganic particles [106,115,116].

#### 1.3.2. Organic Nanoparticles

Cationic lipids or polymers that interact with negatively charged nucleic acids are a mainstay of organic methods. Since multiple formulations of lipid-based drug delivery systems have been approved by the FDA and EMA to deliver different medications, they are among the most appealing non-viral vectors for delivering gene therapies [117]. Following the recent triumph of SARS-CoV-2 vaccinations [118], lipid NPs have gained widespread recognition as genetic material delivery vectors. Because of their positive charge at low pH, the ionizable lipids that make up these spherical vesicles enable contact with nucleic acids by electrostatic forces and endosomal escape once internalized by cells. Furthermore, these lipids are less toxic and immunogenic since they are neutral at physiological pH. Consequently, the first RNAi treatment [119] to be licensed by the FDA can now be delivered thanks to lipid NPs. Lipid nanoparticle methods have also been shown to be capable of delivering CRISPR/Cas9 (clustered regularly interspaced short palindromic repeats-associated protein 9) components to produce clinically meaningful levels of genome editing in vivo.

Extracellular vesicles, or exosomes, range in diameter from 40 to 160 nm and are naturally released by many different types of cells. These vesicles exchange a wide range of cell components, including proteins, RNA, DNA, and other metabolites, across cells to facilitate intercellular communication. Furthermore, a large amount of proteins in their lipid membrane enable more precise targeting and increased stability. However, challenges with manufacturing, separation, and purification impede the utilization of these systems as gene-delivery vectors [117].

Gene therapy delivery through polymer-based methods has also received a lot of research attention. For gene transfection, both natural and synthetic polymers can be utilized. Biocompatible and biodegradable polymers must be chosen carefully when creating NPs [117,120]. The controlled decomposition of biodegradable polymers is an advantage. Once the nanosystem is within the cell, this breakdown releases the plasmid into the cytoplasm of the cell. Additionally, nucleic acids are shielded from nuclease breakdown by polymeric NPs. The nucleic acid can bind to the surface of the nanoparticle due to its cationic nature [106]. When negatively charged nucleic acids interact with positively charged polymers like chitosan or poly-ethylenimine (PEI), NPs known as polyplexes are created. Both polyplexes and lipoplexes have great in vitro transfection efficiency. However, their toxicity and immunogenicity prevent their application in vivo [117,121]. Gene delivery, anti-sense oligonucleotides, and siRNA delivery have all been investigated using dendrimers with positively charged surface groups. Sequence specificity is absent from the dendrimer–gene interaction since it is usually electrostatic. The spherical, nanoscale polymers known as poly (amidoamine) (PAMAM) are one kind of dendrimer that is frequently utilized in gene transfection [122].

**Table 4:** Common nanocarriers for genetic delivery.

Nanocarriers	Formulations	Encapsulated Gene Molecule	Particle Size	Preparatory Techniques	Advantages	References
Lipids	Lipid-like nanomaterials: FTT lipids	Cas9 mRNA and sgRNA	490 nm	Rolling circle amplification (RCA) reaction	Provide cell-type-specific targeting	[123]
	Ionizable lipid cholesterol and the PEGylated lipid	Si RNA and mRNA	~155 and ~125 nm	Microfluidic hydrodynamic focusing and staggered herringbone mixing	Improved gene knockdown ability	[124]
	DLin-MC3-DMA, and DMG-PEG2000	pDNA	400 nm	Ethanol-loading technique	Prolong gene expression	[125]
Polymers	Hyaluronic acid-coated chitosan with AS1411 ligands	Cas9 RNPs	63 nm to 150 nm	Electrostatic adsorption	Improved delivery of CRISPR/Cas9 into the tumor	[126]
	Polyplex	siRNA	25 ± 2 nm	Reversible addition–fragmentation chain transfer (RAFT) polymerization	Deeper penetration of SiRNA Polyplexes into homospheroids	[127]
Inorganic NPs	Gold NPs	SiRNA	42.4 nm	Thiol–gold chemistry	High serum stability and tumor-specific targeting ability	[128]
	Iron oxide NPs	SiRNA	10–20 nm	Co-precipitation method	Efficient delivery of interfering RNA into human embryonic kidney cells (HEK-293); efficient intracellular protein release into the cytosols	[129]
Dendrimers	5 (G5) amine-terminated polyamidoamine (PAMAM) dendrimer	Cas9	100 nm	By reacting 4-(bromomethyl)phenylboronic acid with the dendrimer at different feeding ratios	Efficient intracellular protein release into the cytosols	[130]

#### 1.3.3. Recent Advancement in NP-Based Gene Therapy

Following recent advancement in nanotechnology, various techniques have been employed to improve the functions of NPs for gene therapy, with an emphasis on the targeting property, internalization efficacy, and the cargo release profiles.

#### 1.3.4. Functionalization of NPs

The surface of the NPs needs to be appropriately altered with recognition molecules and immobilized using techniques like glutaraldehyde or carbodiimide in order to enhance active endocytosis. Monoclonal antibodies are the most-often-employed recognition molecules (mAbs). Single-stranded variable fragments (scFv) can be used as an alternative with the same target property and smaller size. NPs functionalized with scFv against HER2 in breast cancer have been shown in in vivo tests to reduce tumor growth. Because of their high cost, it is challenging to utilize Mabs or scFv, making large-scale production techniques difficult [120,131,132].

#### 1.3.5. CRISP/Cas9 Delivery for Genome Editing

A powerful gene-editing tool is CRISPR/Cas9 [133]. The American biologist Jennifer Doudna and the French microbiologist Emmanuelle Charpentier were given the Nobel Prize in Chemistry in October 2020 for “developing a new approach to genome editing.” The greatest hurdle for CRISPR/Cas9 therapy is how to safely and efficiently deliver it to target areas in vivo. However, the nanotechnology-based delivery of CRISPR/Cas9 is paving new ways for cancer gene editing and immunotherapy [133,134]. Liu et al. [135] constructed a multistage delivery nanoparticle (MDNP) for delivering the CRISPR-dCas9 system. They built the core–shell structure. The cationic polymer formed by PEI (polyethyleneimine) NPs modified by phenylboronic acid (PBA) was used as the core. This core was then fused to the plasmid encoding dCas9 and sgRNA. Another polymeric particle described by the same group that created MDNP is called the dual-locking nanoparticle (DLNP). The CRISPR/Cas13a core of DLNPs targets PD-L1 in tumor cells. Once Cas13a reaches tumor cells, it recognizes the PD-L1 mRNA specifically and becomes active [134]. Moreover, Li et al. [136] used human antigen R (HuR) with the CD9 C-terminus to create a novel exosome, which holds great promise for the targeted delivery of CRISPR/dCas9 systems to treat diseases. CRISPR/Cas9 was delivered using both lipid- and polymer-based reagents in clinical trials to treat a variety of disorders.

#### 1.3.6. Use of Cell-Penetrating Peptides

Combinations with cell-penetrating peptides (CPPs) constitute a useful tactic to maximize NPs’ potential under physiological conditions and to optimize their cellular absorption. CPPs are frequently cationic peptide sequences that have been identified as flexible drug delivery vehicles due to their ability to translocate across biological membranes and convey associated cargos into cells. [137]. Different CPPs were added to gelatin-silica NPs, such as a fusogenic peptide made of Tat and influenza hemaglutanin A2, to effectively deliver plasmid DNA with nuclear targeting and endosomal escape characteristics in vivo [138]. Tumor-activatable CPP dual-triggered by reduced pH and matrix metalloproteinase 2 was utilized to label NPs containing siRNA targeting vascular endothelial growth factor and doxorubicin. As a result, the growth of new blood vessels was effectively stopped and the tumor underwent apoptosis [139,140].

#### 1.3.7. Clinical Translation of NP-Based Gene Therapy

In phase-1 clinical trials against cancer, Davis et al. [141] presented the first gene delivery method based on NPs, called CALAA-01. CALAA-01 is composed of a transferrin-targeting ligand that binds to transferrin receptors that are overexpressed on cancer cells, a cyclodextrin-containing polymer, a PEG steric stabilizing agent, and siRNA that targets the M2 subunit of ribonucleotide reductase (RRM2). More recently, a number of lipid-based formulations have been explored in clinical settings. The clinical application of gene–drug combinations, radiation, photodynamic treatment, and immunotherapy has received significant interest lately. A phase-1 study was conducted to evaluate the biological activity and safety of intraperitoneal GEN-1 (IL-12 plasmid formulated with PEG-PEI-cholesterol lipopolymer) administered in combination with standard neoadjuvant chemotherapy for patients with epithelial ovarian, fallopian tube, and primary peritoneal cancer. A phase-2 trial combining oral temozolomide with intravenous SGT-53 (P53 gene therapy) sponsored by SynerGene Therapeutics is being conducted to treat recurrent glioblastoma [109].

### 1.4. Nanomedicine: Application in Tissue Engineering

This strategy for tissue and organ reconstruction first surfaced about thirty years ago [142]. Tissue engineering has been referred to as “an interdisciplinary field which applies the principles of engineering and life sciences towards the development of biological substitutes that aim to maintain, restore or improve tissue function.” Three fundamental techniques are used in this biomedical field to regenerate new tissues: cells, scaffolds, and growth factors [142,143].

Combining tissue engineering and nanomedicine to create the best of both worlds has a significant impact on how people are treated. [144]. Functional tissue and organ replacements necessitate precise spatial and temporal control over biological processes. Key tools for maintaining control over and monitoring the altered tissues are the presentation and regulated local administration of bioactive substances (growth factors, chemokines, inhibitors, cytokines, genes, etc.). Due to this requirement, NP-based systems were used in tissue engineering scaffolds to administer various growth agents, provide contrast for imaging, and regulate the scaffolds’ characteristics. Materials such as polymers, metals, ceramics, and their various composites can be used to produce NPs depending on the application, as shown in Table 5 [145]. Moreover, the ECM that surrounds cells in tissues is made up of a naturally occurring network of nanofibers arranged hierarchically. Recently, biomimetic microenvironments may have been designed and created at the nanoscale thanks to recent developments in nanotechnology, offering a native ECM analog. These technologies have been notably used to produce nanofeatured scaffolds and nanotopographic surfaces, as well as to encapsulate and regulate the spatiotemporal release of medications (such as growth factors). Consequently, these nanodevices provide a way to control a variety of cellular processes, such as gene expression and cell adhesion [146].

#### 1.4.1. Advanced Characteristics of NPs Assisting in Tissue Engineering

Tissue engineering NPs have sophisticated properties that make them useful in a range of applications. By modifying their mechanical, electrical, and biological characteristics, these NPs can be made to resemble the microenvironment unique to a given tissue [158]. Additionally, they can be made to go past various delivery barriers, making it possible to deliver drugs to specific tissues effectively [159]. Moreover, engineered NPs can be tuned to fulfill specific performance goals in a variety of engineering applications thanks to their extensive spectrum of physicochemical properties, including electronic, optical, magnetic, mechanical, thermal, vibrational, and surface properties [160] In addition, biomedical polymers, bioceramics, and other inorganic materials are to be mixed for superior qualities in order to imitate the natural tissue structure. Composites of polymer–inorganic NPs can serve as scaffolds for bone that has been tissue-engineered. Nanoparticle-filled polymers are superior to traditional polymer composites in many ways, including reduced weight, enhanced mechanical qualities, increased durability, and a bioactive interface—a crucial component for the successful and long-term use of prostheses. Data indicate that in order to promote better regeneration, nanostructured composites may be able to mimic the surface and/or chemical characteristics of bone and cartilage, respectively [161]. Thus, depending on the applications, using the appropriate kind of NPs in TE can greatly improve the scaffolds’ mechanical, electrical, and biological qualities, in addition to serving a variety of other purposes.

#### 1.4.2. Enhanced Metallic Properties

The enhanced characteristics of metallic NPs aid in tissue engineering. The benefits of these NPs include increased proliferation and adherence of cells, regulated growth factor release, and non-invasive tissue regeneration process monitoring [162]. To improve their qualities and promote tissue regeneration, they can be included in scaffolds for tissue engineering [163]. For a variety of tissue regeneration applications, such as cardiac, bone, neural, and skin tissue engineering, metallic NPs, as well as other nanoparticle kinds like carbon and ceramic NPs, are being investigated [164]. Yue et al. developed dual stimuli-responsive hydrogels with interpenetrating polymer networks via the crosslinking of N-isopropyl acrylamide (NIPAM) and sodium alginate (SA). 2,2,6,6-tetramethyl-piperidine-1-oxyl-oxidized cellulose nanofibers (TOCNF) derived from energy cane bagasse as a carrier material for nanosilicas (NS) and nanoclays (NC). The hydrogels are dual stimuli-responsive and have an interpenetrating polymer network structure. Out of all these hydrogels, PNIPAM/SA-TOCNF-NS has the highest compressive strength, measuring 66.7 kPa, which is 5.65 times greater than PNIPAM/SA [165]. Furthermore, for the purpose of promoting neuronal growth and differentiation, nerve growth factor (NGF)-decorated superparamagnetic iron oxide (SPIO)–Au core–shell NPs [166] with minimal toxicity have recently been produced. Applying an external magnetic field has also been utilized to regulate the orientation of collagen fibers remotely and dynamically in situ during the gelation phase using magnetic NPs. Three-dimensional gel neurons stimulated by magnetism exhibited cellular survival, spontaneous electrical activity, and an elongated, co-oriented form. Iron oxides are helpful in conjugating different peptides and growth factors to rejuvenate and rebuild brain tissue since they can also cross the blood–brain barrier [145]. The multifunctional BG (bioactive glass) NPs [167] based on the poly-citrate-siloxane elastomer are utilized as a useful substance for bone tissue regeneration.

#### 1.4.3. Enhanced Electrical Properties

In tissue engineering, the enhanced electrical characteristics of NPs are useful because they may create conductive scaffolds that resemble the extracellular milieu of the body and encourage cellular activity and healing [168]. The concentration and shape of the NPs can be changed to modify the electrical characteristics of these scaffolds. Electroconductive biomaterials, such as NPs, can be used in 3D bioprinting to direct the healing of various tissue types and affect the fate of cells [169,170]. It has been demonstrated that electrical stimulation promotes neuron regeneration. Graphene (G)-based materials operate well in neural tissue engineering due to their high electrical conductivity, flexibility, and mechanical strength. G-based materials can also speed up the differentiation and proliferation of neuronal cells. Furthermore, carbon nanotubes have emerged as one of the most-often-used materials in brain tissue creation due to their morphology resembling that of neurites. Also, it has been demonstrated that a carbon nanotube with dendrites of comparably small size has greater promise for treating neuropathy and damage to nerve tissue. It also improves the prospect of investigating, mending, and thrilling brain networks. They are also very promising in other technical fields like sensors and conductive composite materials because to their good mechanical, thermal, and electrical qualities [164]. Additionally, gold nanowires have been employed as conductive materials to improve the electrical interaction between the cells. Synapse development occurred as heart muscle cells gradually began to proliferate inside the three-dimensional porous scaffolds. Research has also demonstrated that adding carbon nanotubes (CNTs) to polymer composites can greatly increase the conductivity and support the functions of cardiomyocytes [171]. Thus, the overall goal of developing sophisticated tissue engineering techniques to replace or repair damaged tissues is aided by the improved electrical characteristics of NPs.

#### 1.4.4. Enhanced Biological Properties

Tissue engineering relies heavily on the sophisticated biological characteristics of NPs. Tissue engineering can benefit from the advantages that NPs provide, including contrasting agent qualities, scalable features, and decreased toxicity. Numerous substances and materials, such as polymers, ceramics, carbon nanotubes, graphene, fullerenes, and quantum dots, can be used to create them [162,163]. Additionally, gold nanoparticles (GNPs) have been shown to stimulate the osteogenic development of the osteoblast precursor cell line MC3T3-E1 in bone TE. Furthermore, these NPs protected osteoblastic cells from mitochondrial dysfunction while also influencing the generation of osteoclasts, or bone-resorbing cells, from hematopoietic cells. However, it was discovered that the size of GNPs influences this osteogenic differentiation. While one study found that GNPs with diameters between 30 and 50 nm were more beneficial and efficient for human adipose-derived stem cell (ADSC) activity, another study found that MC3T3-E1 osteoblast-like cells preferred GNPs with diameters between 20 and 40 nm [171]. On the other hand, NPs, such as superparamagnetic iron oxide NPs (SPIONs), have been shown to promote osteoblast proliferation, differentiation, and angiogenesis in bone tissue engineering [172].

#### 1.4.5. Three-Dimensional and Four-Dimensional Nano Printing

The development of three-dimensional (3D) bioprinting techniques has made it possible to create pre-programmed synthetic tissues that exhibit precise geometries and have controlled cellular composition and spatial distribution. With the potential to improve results, new bioinks with electroconductive qualities may be able to direct the repair of different kinds of tissues, such as bone, heart, and nerve tissue, by influencing cellular destinies and functions [169]. Furthermore, polymeric hydrogels are being explored extensively as ink materials for 3D and 4D bioprinting applications due to their ability to be printed into scaffolds with a hierarchical structure and intrinsic properties resembling those of the original tissue’s extracellular matrix. By incorporating nanoscale material additives such as NPs into the ink composition, it is now possible to significantly tune the mechanical, biological, structural, and physicochemical properties of the material during and after printing [173].

#### 1.4.6. Selective Cell Attachment

Scaffold surfaces can now be modified chemically or via hyaluronan (HA), poly(ethylene oxide) (Pluronics), PEG, glycocalyx, anti-bacterial coatings comprising silver or N-alkylated poly(vinylpyridine), bioadhesive coatings comprising RGD peptide insertion, growth factor attachment, decoration with other bioactive groups, plasma etching, or other means to achieve desired cell adhesion but repel unwanted attachment [161].

#### 1.4.7. Advanced Nanocomposite in Tissue Engineering

Organ physiology encompasses many individual intricate biological systems, often making the development of new regenerative medicine strategies quite challenging as they have to adopt a more holistic perspective to cope with the requirements that precise medicine imposes. Organ-on-a-chip technology is a relatively new field based on the combination of various biological, chemical, and engineering advancements to create miniature-like structures that resemble the microenvironment of the native tissue. Its main concept revolves around the integration of different microfluidic chips as parts of polymeric constructs that can manipulate cell behavior at close proximity and thus mimic small-scale physiological processes. Because the main ingredient of microchips is silicon, organ-on-a-chip structures can be considered as nanocomposites [173].

#### 1.4.8. Smart Scaffolds

A fundamental function of living systems is their response to stimuli. Scientists have been creating tissue engineering scaffolds that react to external stimuli, including temperature, pH, light, electric field, magnetic field, chemicals, and ionic strength, by incorporating traits from biological systems found in nature. Shape/position, structure, surface features, solubility, integrated sensing, actuation (secretion), the development of a complex molecular self-assembly, or a sol-to-gel transition are some examples of these responses [161].

Biomimetic smart materials are an important type of smart materials. The structure, function, and production of biological materials serve as biological inspiration for their development. Smart scaffolds can elicit the optimal cell responses because stem cells in touch with the scaffold can sense many qualities, such as stiffness and nanostructure, and respond appropriately [174].

### 1.5. Nanomedicine in Vaccine’s Developments

Vaccines represent a cornerstone of public health interventions, remarkably reducing morbidity and mortality from infectious diseases. However, similar to other medical advancements, vaccines are not without limitations. These limitations include suboptimal immunogenicity, non-specific delivery, and rapid degradation, all of which can hinder vaccine efficacy [175]. The field of nanomedicine offers a strategic approach to addressing these limitations and achieving near-optimal vaccination strategies. NPs can be engineered to encapsulate vaccine antigens, improving their stability and facilitating targeted delivery to immune cells. This targeted delivery enhances antigen presentation and immune response, potentially leading to more effective vaccines [176]. The rapid development and deployment of COVID-19 vaccines exemplify the transformative potential of nanomedicine in vaccine design. Several approved COVID-19 vaccines utilize cationic lipid NPs for mRNA delivery, carrying the genetic instruction for intracellular production of the COVID spike(S) protein, a foreign mRNA that would readily disintegrate without the protective nano shell [177].

**Figure 2:**
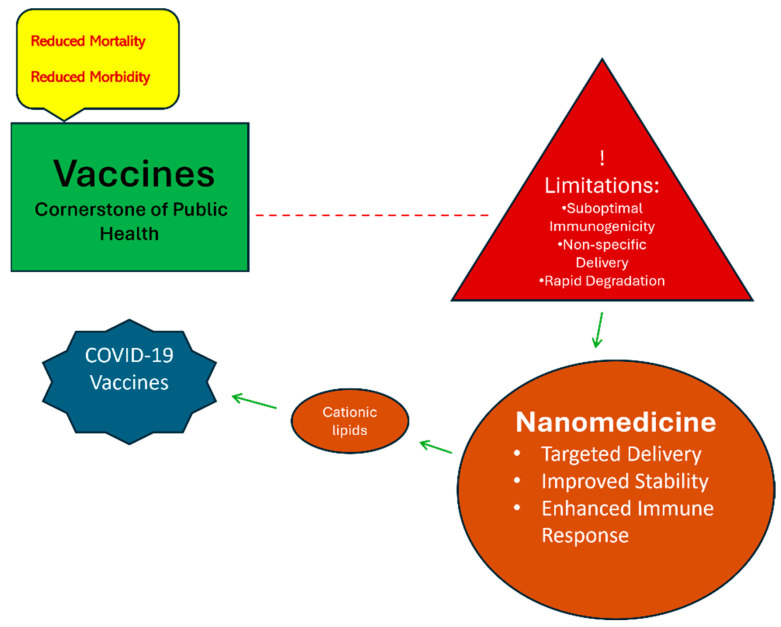
The value of nanomedicine in vaccine development with special emphasis on COVID vaccine development.

The success of these vaccines underscores the significant role nanomedicine can play in future vaccine development (Figure 2).

The following are some of the properties that can be bestowed on vaccines by NPs.

#### 1.5.1. Encapsulation and Protection of Vaccine Antigens

Traditional vaccines usually employ weak or inactivated pathogens or purified protein antigens. These antigens can be susceptible to degradation by enzymes and harsh physiological conditions before reaching immune cells [178]. NPs can be designed with a biocompatible shell that encapsulates these antigens, protecting them from degradation and premature release. This allows for the sustained and targeted delivery to the desired immune compartment [179].

Messenger RNA (mRNA) vaccines are now a main focus in vaccine development due to their ability to rapidly induce robust immune responses. However, unprotected mRNA is highly unstable in vivo [180]. Lipid NPs (LNPs) have emerged as a powerful platform for mRNA delivery. These LNPs encapsulate mRNA molecules within their hydrophobic core, shielding them from enzymatic degradation and facilitating their delivery to antigen-presenting cells (APCs). A study by Arevalo et al. [181] demonstrated that LNP-encapsulated mRNA, encoding influenza virus hemagglutinin (HA) antigens from an array of influenzas virus subtypes, elicited a significantly stronger immune response compared to free mRNA, highlighting the protective role of NPs.

#### 1.5.2. Co-Delivery of Antigens and Adjuvants

Traditional adjuvants, such as aluminum salts, enhance the immune response to co-administered antigens but often induce side effects [182]. Nanoparticle design allows for the co-encapsulation of antigens and adjuvants within a single carrier. This targeted co-delivery system delivers both components simultaneously to APCs, leading to a more potent and controlled immune response [176].

Stimulating pattern recognition receptors (PRRs): NPs can be engineered with specific surface modifications to interact with PRRs on APCs, such as Toll-like receptors (TLRs). TLR activation by adjuvants encapsulated within NPs triggers the release of pro-inflammatory cytokines and co-stimulatory molecules, leading to enhanced antigen presentation and T cell activation [183]. For instance, Stickdorn et al. [184] showed that cationic polymeric NPs loaded with ovalbumin antigen and a TLR7/8 agonist adjuvant resulted in superior antigen-specific CD8+ T cell responses compared to free antigen or antigen with a separate adjuvant.

Furthermore, with the possible combined effects of antigen protection, targeted delivery, and the co-delivery of adjuvants with NPs, a synergistic response is highly likely. This synergy would lead to a more potent and specific immune response compared to free antigens or traditional vaccines.

#### 1.5.3. Targeted Delivery to Immune Cells

NPs can be further engineered to target specific immune cell populations by incorporating ligands that bind to receptors on these cells. This targeted delivery approach reduces off-target effects and optimizes antigen presentation, leading to a more focused and efficient immune response [185]. Table 6 shows the ongoing research on enhancing vaccinations using different NPs.

## 2. Challenges and Future Perspective

Nanomaterials play a significant role in therapeutic engineering (TE), offering unique advantages in enhancing biological, mechanical, and electrical properties, as well as exhibiting anti-microbial effects, enabling gene delivery, and facilitating the creation of engineered tissues. Despite these advancements, several critical obstacles must first be overcome to realize widespread clinical adoption. One of the key challenges in the utilization of nanomaterials for therapeutic applications is the need for robust tools and procedures to assess their safety profiles. Although most NPs exhibit much-improved safety profiles and fewer side effects, NPs may have complex interactions with biological systems, and their toxicity, carcinogenicity, and teratogenicity must be thoroughly evaluated. Recent studies have highlighted dose- and exposure-dependent relationships in the toxicity of NPs, underscoring the importance of comprehensive risk assessment [189,190]. Despite the increasing prevalence of nanoparticle-containing products, there remain methodological and scientific gaps in our understanding of the specific risks associated with different nanomaterials [171]. Therefore, advancements in nanotoxicology research are essential for developing better safety assessment methods. This includes the refinement of in vitro and in vivo models to simulate realistic exposure scenarios and predict nanoparticle behavior in biological environments [191,192].

In addition, with the industrial scale-up of advanced nanomaterials for therapeutic applications, attention must be focused on the long-term effects of chronic nanoparticle exposure. Continuous exposure to NPs over extended periods raises concerns regarding cumulative toxicity and potential health impacts [189,190]. Addressing these challenges requires a concerted effort to develop standardized protocols for assessing the chronic effects of NPs and establishing safety guidelines for their use in clinical settings [193]. In this regard, establishing a robust regulatory framework is imperative to ensure the safe integration of nanomaterials into clinical applications. Collaborative efforts among regulatory agencies, researchers, and industry stakeholders are needed in order to address knowledge gaps and define standardized safety guidelines.

## 3. Conclusions

Nanomaterials are playing a significant role in the advancement of biological and medical applications in this new era. They enable the realization of previously unsolved mysteries and seemingly impossible therapies by offering improved biocompatibility, controlled drug release, precise targeting capabilities, and a large surface area conducive to interactions with biological systems. Particularly, the field of theranostics is progressing rapidly with the integration of nanotechnology. Looking ahead, we must overcome the remaining barriers preventing the clinical translation of nanomedicine while addressing toxicity concerns associated with nanomaterials. By doing so, we can fully leverage the diverse properties of nanomaterials discussed in this article to advance healthcare and biomedical technologies.

## Figures and Tables

**Table 1:** Examples of anti-cancer nanomedicines.

Drug Name	Platform	API	Approval and Clinical Trial Status
SMANCS	Polymer conjugate	Neocarzinostatin	Approved (1993), Japan
Doxil^®^	Liposome	Doxorubicin	Approved (1995), USA
DaunoXome^®^	Liposome	Doxorubicin	Approved (1996), USA
Ontak^®^	Fusion protein	Diphtheria toxin	Approved (1999), USA
Myocet^®^	Liposome	Doxorubicin	Approved (2000), Europe
Zevalin^®^	Anti-CD20 antibody (ADC^#^)	Yttrium-90	Approved (2002), USA
Abraxane^®^	Albumin-bound nanoparticle	Paclitaxel	Approved (2005), USA
Genexol^®^-PM	Polymeric micelle	Paclitaxel	Approved (2007), Korea
Marqibo^®^	Liposome	Vincristine	Approved (2012), USA
Onivyde^®^	Liposome	Irinotecan	Approved (2015), USA
Vyxeos^®^	Liposome	Daunorubicin and cytarabine	Approved (2017), USA
Mylotarg^®^	Anti-CD33 antibody (ADC)	Calicheamicin	Approved (2017), USA
Hensify^®^	Nanoparticle	Hafnium oxide	Approved (2019), USA
CPC634	Polymeric micelle	Docetaxel	In clinical phase II trial
NC-4016	Polymeric micelle	Oxaliplatin	In clinical phase I trial
NK105	Micelle	Paclitaxel	In clinical phase III trial
NC-6004	Micelle	Cisplatin	In clinical phase II trial

Sources: FDA website, original publications, reviews, and websites of pharmaceutical companies supplying and developing these drugs). ADC: antibody–drug conjugate.

**Table 2:** Application and therapeutic strategies of NPs.

Category	Mechanism	Subcategory	Typical Examples
PDT	Specific photosensitizers are used to produce active oxidative substances under light irradiation of specific wavelengths, thereby triggering the death of cancer cells or other abnormal tissues.	PNPSs	HPMA [27]
		Nanoliposomes	RALP@HOC@Fe_3_O_4_ [46]
		Nanohydrogel particles	HA-ADH-PpIX [47]
		MNPSs	GNRs@mSiO_2_ [48] B-TiO_2_@SiO_2_–HA [49]
		CNPSs	single-walled carbon nanotubes (SWNTs) [50] HA-TiO2-GO153 [51] amino-N-GQDs [52]
		QDPSs	CdTe QDs [53]
		UCNPs	OP-UCNPs-C [54]
		Organic frameworks	MOFs [55] COFs [56],
PTT	By using nanomaterials that absorb light of specific wavelengths, local heat is generated under light irradiation, thereby triggering thermal damage and the death of cancer cells or abnormal tissues.	Metal nanoparticles	PEG-modified gold nanorods [57] Copper sulfide (CuS) [58] MGNPs [59] Au NBPs@PDA [60] Bi@ZIF-8 [61] LV-TAX/Au@Ag [62]
		Carbon nanoparticles	GO-PEG-Ce6 [63] C-dots@GNR [64] rGO-FA [61] FA-SWCNT [65]
		Quantum dots	BPQDs [66]
PDD	Tumors or other lesions are detected using the fluorescent signal produced by a photosensitizer under irradiation with light of a specific wavelength. When the photosensitizer is activated at a specific wavelength, it emits fluorescence, allowing for the diagnosis of lesions.	Metal nanoparticles	ZnPcS4 AuNP-S-PEG5000-NH_2_ Anti-GCC mAb [67]

Abbreviations: polymer-modified nanophotosensitizers (PNPSs); metal nanoclusters-based photosensitizers (MNPSs); carbon nanophotosensitizers (CNPSs); quantum dot photosensitizer (QDPSs); upconversion nanoparticles (UCNPs); metal–organic frameworks (MOFs); covalent organic frameworks (COFs); photothermal therapy (PTT); photodynamic diagnosis (PDD).

**Table 5:** Various recent nanocomposites available for tissue engineering.

Tissue Engineering Field	NPs Involved in Scaffold	Application and Advantages	References
Cardiovascular tissue engineering	Carbon nanotubes	Increased the length of cells with improved biocompatibility, making them an ideal option for vessel construction in tissue engineering	[147]
	poly (L-lactide-co-caprolactone) nanofibers	Reduced aortic inflammation and encourage aortic remodeling following the implantation of a stent–graft	[148]
Dental tissue engineering	Monodispersed silica NPs (SNPs)	Provided a safe and effective plateform for the creation of sustainable anti-biofouling surfaces for dental implants and other biomedical devices	[149]
	Polycaprolactone (PCL)-based block copolymers	Enhanced the effectiveness of antibiotics’ anti-biofilm properties, addressing the serious risk posed by biofilm-associated infections such as periodontitis.	[150]
Skin tissue engineering	Alginate–polyvinyl alcohol (PVA) nanofiber	Showed a fiber diameter of about 172,242–326,244 n with improved tensile strength, thus qualifying the fiber to be used in wound dressing	[151]
	Collagen/poly(l-lactic acid)-co-PCL nanofiber mesh	Improved the cell’s capacity to disseminate, remain viable, and adhere, and could potentially be used as a material for tissue-engineered vascular grafts	[152]
	AgNPs hydrogel	Accelerated skin tissue regeneration and provide effective care in chronic wounds	[153]
Neural tissue engineering	Chitosan/polyethylene glycol/multiwalled carbon nanotube composite	Enhanced the elastic modulus of the scaffold and expressed nerve growth receptor in the scaffold for neural tissue engineering	[154]
	PCL/gelatin nanofibrous scaffold	Could be used as electrically conductive scaffolds along with anti-bacterial properties in neural tissue engineering	[155]
Bone tissue engineering	Graphene oxide loaded magnetic nanoparticle	Scaffold promoted the development of bone mesenchymal stem cells and improve biological functions	[156]
	Cholesterol-bearing pullulan (CHP) nanogel	Stimulated bone morphogenetic protein (BMP)-2-induced local bone formation	[157]

**Table 6:** Ongoing research on enhancing vaccinations using different NPs.

Vaccine Target	Nanoparticle Platform	Delivery Mechanism	Reference
HIV	Lipid-based	Encapsulate viral antigens and adjuvants for targeted delivery to antigen-presenting cells (APCs)	[186]
Influenza	Liposomes	Encapsulate viral antigens and adjuvants to promote cellular uptake of the antigen payload	[187]
COVID-19	Cationic lipids	Encapsulate mRNA encoding viral antigens; cationic lipids facilitate cellular uptake of the mRNA for translation into viral proteins, triggering an immune response	[188]

## References

[1] Haleem A., Javaid M., Singh R.P., Rab S., Suman R. (2023). Applications of nanotechnology in medical field: A brief review. Glob. Health J..

[2] Greish K. (2018). Recent and future advances in anticancer drug delivery: An interview with Khaled Greish. Ther. Deliv..

[3] Ramos A.P., Cruz M.A.E., Tovani C.B., Ciancaglini P. (2017). Biomedical applications of nanotechnology. Biophys. Rev..

[4] Park W., Heo Y.-J., Han D.K. (2018). New opportunities for nanoparticles in cancer immunotherapy. Biomater. Res..

[5] Debele T.A., Yeh C.F., Su W.P. (2020). Cancer Immunotherapy and Application of Nanoparticles in Cancers Immunotherapy as the Delivery of Immunotherapeutic Agents and as the Immunomodulators. Cancers.

[6] Matsumura Y., Maeda H. (1986). A new concept for macromolecular therapeutics in cancer chemotherapy: Mechanism of tumoritropic accumulation of proteins and the antitumor agent smancs. Cancer Res..

[7] Fang J., Islam W., Maeda H. (2020). Exploiting the dynamics of the EPR effect and strategies to improve the therapeutic effects of nanomedicines by using EPR effect enhancers. Adv. Drug Deliv. Rev..

[8] Islam R., Maeda H., Fang J. (2022). Factors affecting the dynamics and heterogeneity of the EPR effect: Pathophysiological and pathoanatomic features, drug formulations and physicochemical factors. Expert Opin. Drug Deliv..

[9] Maeda H. (2010). Tumor-selective delivery of macromolecular drugs via the EPR effect: Background and future prospects. Bioconjugate Chem..

[10] Maeda H. (2015). Toward a full understanding of the EPR effect in primary and metastatic tumors as well as issues related to its heterogeneity. Adv. Drug Deliv. Rev..

[11] Maeda H., Nakamura H., Fang J. (2013). The EPR effect for macromolecular drug delivery to solid tumors: Improvement of tumor uptake, lowering of systemic toxicity, and distinct tumor imaging in vivo. Adv. Drug Deliv. Rev..

[12] Maeda H., Tsukigawa K., Fang J. (2016). A retrospective 30 years after discovery of the enhanced permeability and retention effect of solid tumors: Next-generation chemotherapeutics and photodynamic therapy—Problems, solutions, and prospects. Microcirculation.

[13] Ding Y., Xu Y., Yang W., Niu P., Li X., Chen Y., Li Z., Liu Y., An Y., Liu Y. (2020). Investigating the EPR effect of nanomedicines in human renal tumors via ex vivo perfusion strategy. Nano Today.

[14] Lee H., Shields A.F., Siegel B.A., Miller K.D., Krop I., Ma C.X., LoRusso P.M., Munster P.N., Campbell K., Gaddy D.F. (2017). 64Cu-MM-302 positron emission tomography quantifies variability of enhanced permeability and retention of nanoparticles in relation to treatment response in patients with metastatic breast cancer. Clin. Cancer Res..

[15] Maeda H., Sawa T., Konno T. (2001). Mechanism of tumor-targeted delivery of macromolecular drugs, including the EPR effect in solid tumor and clinical overview of the prototype polymeric drug SMANCS. J. Control. Release.

[16] Germain M., Caputo F., Metcalfe S., Tosi G., Spring K., Åslund A.K., Pottier A., Schiffelers R., Ceccaldi A., Schmid R. (2020). Delivering the power of nanomedicine to patients today. J. Control. Release.

[17] Lammers T., Ferrari M. (2020). The success of nanomedicine. Nano Today.

[18] Martins J.P., Das Neves J., de la Fuente M., Celia C., Florindo H., Günday-Türeli N., Popat A., Santos J.L., Sousa F., Schmid R. (2020). The solid progress of nanomedicine. Drug Deliv. Transl. Res..

[19] Lin L., Song X., Dong X., Li B. (2021). Nano-photosensitizers for enhanced photodynamic therapy. Photodiagnosis Photodyn. Ther..

[20] Islam W., Matsumoto Y., Fang J., Harada A., Niidome T., Ono K., Tsutsuki H., Sawa T., Imamura T., Sakurai K. (2021). Polymer-conjugated glucosamine complexed with boric acid shows tumor-selective accumulation and simultaneous inhibition of glycolysis. Biomaterials.

[21] Kim A., Suzuki M., Matsumoto Y., Fukumitsu N., Nagasaki Y. (2021). Non-isotope enriched phenylboronic acid-decorated dual-functional nano-assembles for an actively targeting BNCT drug. Biomaterials.

[22] Cross R. (2021). Without these lipid shells, there would be no mRNA vaccines for COVID-19. Chem. Eng. News.

[23] Amaravadi R.K., Kimmelman A.C., Debnath J. (2019). Targeting Autophagy in Cancer: Recent Advances and Future Directions. Cancer Discov..

[24] Zhang J., Jiang C., Figueiro Longo J.P., Azevedo R.B., Zhang H., Muehlmann L.A. (2018). An updated overview on the development of new photosensitizers for anticancer photodynamic therapy. Acta Pharm. Sin. B.

[25] Alsaab H.O., Alghamdi M.S., Alotaibi A.S., Alzhrani R., Alwuthaynani F., Althobaiti Y.S., Almalki A.H., Sau S., Iyer A.K. (2020). Progress in Clinical Trials of Photodynamic Therapy for Solid Tumors and the Role of Nanomedicine. Cancers.

[26] Pham T.C., Nguyen V.N., Choi Y., Lee S., Yoon J. (2021). Recent Strategies to Develop Innovative Photosensitizers for Enhanced Photodynamic Therapy. Chem. Rev..

[27] Islam R., Kotalik K., Subr V., Gao S., Zhou J.R., Yokomizo K., Etrych T., Fang J. (2023). HPMA copolymer conjugated 5-aminolevulinic acid exhibits superior efficacy for photodynamic therapy with tumor-responsive and targeting properties. Nanomedicine.

[28] Xiang Y., Zheng S., Yuan S., Wang J., Wu Y., Zhu X. (2022). Near-infrared mediated orthogonal bioimaging and intracellular tracking of upconversion nanophotosensitizers. Mikrochim. Acta.

[29] Choi S.H., Kim K.H., Song K.H. (2017). Effect of Methyl Aminolevulinate Photodynamic Therapy With and Without Ablative Fractional Laser Treatment in Patients With Microinvasive Squamous Cell Carcinoma: A Randomized Clinical Trial. JAMA Dermatol..

[30] Chi Y.F., Qin J.J., Li Z., Ge Q., Zeng W.H. (2020). Enhanced anti-tumor efficacy of 5-aminolevulinic acid-gold nanoparticles-mediated photodynamic therapy in cutaneous squamous cell carcinoma cells. Braz. J. Med. Biol. Res..

[31] Shi H., Sadler P.J. (2020). How promising is phototherapy for cancer?. Br. J. Cancer.

[32] Van Doeveren T.E.M., Karakullukcu M.B., van Veen R.L.P., Lopez-Yurda M., Schreuder W.H., Tan I.B. (2018). Adjuvant photodynamic therapy in head and neck cancer after tumor-positive resection margins. Laryngoscope.

[33] Ericson M.B., Wennberg A.M., Larkö O. (2008). Review of photodynamic therapy in actinic keratosis and basal cell carcinoma. Ther. Clin. Risk Manag..

[34] Leon D., Buchegger K., Silva R., Riquelme I., Viscarra T., Mora-Lagos B., Zanella L., Schafer F., Kurachi C., Roa J.C. (2020). Epigallocatechin Gallate Enhances MAL-PDT Cytotoxic Effect on PDT-Resistant Skin Cancer Squamous Cells. Int. J. Mol. Sci..

[35] Akopov A., Papayan G. (2021). Photodynamic theranostics of central lung cancer: Present state and future prospects. Photodiagnosis Photodyn. Ther..

[36] Banerjee S.M., MacRobert A.J., Mosse C.A., Periera B., Bown S.G., Keshtgar M.R.S. (2017). Photodynamic therapy: Inception to application in breast cancer. Breast.

[37] Qiao L., Yang H., Shao X.X., Yin Q., Fu X.J., Wei Q. (2022). Research Progress on Nanoplatforms and Nanotherapeutic Strategies in Treating Glioma. Mol. Pharm..

[38] Kim H.S., Lee D.Y. (2022). Nanomedicine in Clinical Photodynamic Therapy for the Treatment of Brain Tumors. Biomedicines.

[39] Sun D., Zhang Z., Chen M., Zhang Y., Amagat J., Kang S., Zheng Y., Hu B., Chen M. (2020). Co-Immobilization of Ce6 Sono/Photosensitizer and Protonated Graphitic Carbon Nitride on PCL/Gelation Fibrous Scaffolds for Combined Sono-Photodynamic Cancer Therapy. ACS Appl. Mater. Interfaces.

[40] Zhuo X., Liu Z., Aishajiang R., Wang T., Yu D. (2023). Recent Progress of Copper-Based Nanomaterials in Tumor-Targeted Photothermal Therapy/Photodynamic Therapy. Pharmaceutics.

[41] Luo H., Gao S. (2023). Recent advances in fluorescence imaging-guided photothermal therapy and photodynamic therapy for cancer: From near-infrared-I to near-infrared-II. J. Control. Release.

[42] Di Corato R., Béalle G., Kolosnjaj-Tabi J., Espinosa A., Clément O., Silva A.K.A., Ménager C., Wilhelm C. (2015). Combining magnetic hyperthermia and photodynamic therapy for tumor ablation with photoresponsive magnetic liposomes. ACS Nano.

[43] Liao S., Cai M., Zhu R., Fu T., Du Y., Kong J., Zhang Y., Qu C., Dong X., Ni J. (2023). Antitumor Effect of Photodynamic Therapy/Sonodynamic Therapy/Sono-Photodynamic Therapy of Chlorin e6 and Other Applications. Mol. Pharm..

[44] Xin J., Wang S., Wang J., Fu L., Zhang Z., Yao C. (2022). A Nucleus-Targeted Nanosystem Integrated with Photodynamic Therapy and Chemotherapy. J. Biomed. Nanotechnol..

[45] Babic A., Herceg V., Bastien E., Lassalle H.P., Bezdetnaya L., Lange N. (2018). 5-Aminolevulinic Acid-Squalene Nanoassemblies for Tumor Photodetection and Therapy: In Vitro Studies. Nanoscale Res. Lett..

[46] Zhao Z., Wang W., Li C., Zhang Y., Yu T., Wu R., Zhao J., Liu Z., Liu J., Yu H. (2019). Reactive Oxygen Species–Activatable Liposomes Regulating Hypoxic Tumor Microenvironment for Synergistic Photo/Chemodynamic Therapies. Adv. Funct. Mater..

[47] Xu X., Zeng Z., Huang Z., Sun Y., Huang Y., Chen J., Ye J., Yang H., Yang C., Zhao C. (2020). Near-infrared light-triggered degradable hyaluronic acid hydrogel for on-demand drug release and combined chemo-photodynamic therapy. Carbohydr. Polym..

[48] Luo L., Sun W., Feng Y., Qin R., Zhang J., Ding D., Shi T., Liu X., Chen X., Chen H. (2020). Conjugation of a Scintillator Complex and Gold Nanorods for Dual-Modal Image-Guided Photothermal and X-ray-Induced Photodynamic Therapy of Tumors. ACS Appl. Mater. Interfaces.

[49] Guo X., Wen C., Xu Q., Ruan C., Shen X.C., Liang H. (2021). A full-spectrum responsive B-TiO_2_@SiO_2_-HA nanotheranostic system for NIR-II photoacoustic imaging-guided cancer phototherapy. J. Mater. Chem. B.

[50] Murakami T., Nakatsuji H., Inada M., Matoba Y., Umeyama T., Tsujimoto M., Isoda S., Hashida M., Imahori H. (2012). Photodynamic and photothermal effects of semiconducting and metallic-enriched single-walled carbon nanotubes. J. Am. Chem. Soc..

[51] Ding Y., Zhou L., Chen X., Wu Q., Song Z., Wei S., Zhou J., Shen J. (2016). Mutual sensitization mechanism and self-degradation property of drug delivery system for in vitro photodynamic therapy. Int. J. Pharm..

[52] Kuo W.S., Shao Y.T., Huang K.S., Chou T.M., Yang C.H. (2018). Antimicrobial Amino-Functionalized Nitrogen-Doped Graphene Quantum Dots for Eliminating Multidrug-Resistant Species in Dual-Modality Photodynamic Therapy and Bioimaging under Two-Photon Excitation. ACS Appl. Mater. Interfaces.

[53] Sun J., Guo Y., Zhu L., Yang L., Shi W., Wang K., Zhang H. (2017). Photodynamic Therapy of Human Hepatoma Using Semiconductor Quantum Dots as Sole Photosensitizer. Part. Part. Syst. Charact..

[54] Zhang Z., Jayakumar M.K.G., Shikha S., Zhang Y., Zheng X., Zhang Y. (2020). Modularly Assembled Upconversion Nanoparticles for Orthogonally Controlled Cell Imaging and Drug Delivery. ACS Appl. Mater. Interfaces.

[55] Liu W., Wang Y.M., Li Y.H., Cai S.J., Yin X.B., He X.W., Zhang Y.K. (2017). Fluorescent Imaging-Guided Chemotherapy-and-Photodynamic Dual Therapy with Nanoscale Porphyrin Metal-Organic Framework. Small.

[56] Zhang L., Wang S., Zhou Y., Wang C., Zhang X.Z., Deng H. (2019). Covalent Organic Frameworks as Favorable Constructs for Photodynamic Therapy. Angew. Chem. Int. Ed. Engl..

[57] Niidome T., Yamagata M., Okamoto Y., Akiyama Y., Takahashi H., Kawano T., Katayama Y., Niidome Y. (2006). PEG-modified gold nanorods with a stealth character for in vivo applications. J. Control Release.

[58] Li Y., Lu W., Huang Q., Li C., Chen W. (2010). Copper sulfide nanoparticles for photothermal ablation of tumor cells. Nanomedicine.

[59] Elbialy N.S., Fathy M.M., Al-Wafi R., Darwesh R., Abdel-Dayem U.A., Aldhahri M., Noorwali A., Al-Ghamdi A.A. (2019). Multifunctional magnetic-gold nanoparticles for efficient combined targeted drug delivery and interstitial photothermal therapy. Int. J. Pharm..

[60] Wang J., Gao Y., Liu P., Xu S., Luo X. (2020). Core-Shell Multifunctional Nanomaterial-Based All-in-One Nanoplatform for Simultaneous Multilayer Imaging of Dual Types of Tumor Biomarkers and Photothermal Therapy. Anal. Chem..

[61] Mun S.G., Choi H.W., Lee J.M., Lim J.H., Ha J.H., Kang M.J., Kim E.J., Kang L., Chung B.G. (2020). rGO nanomaterial-mediated cancer targeting and photothermal therapy in a microfluidic co-culture platform. Nano Converg..

[62] Wang K., Cai Z., Fan R., Yang Q., Zhu T., Jiang Z., Ma Y. (2020). A tumor-microenvironment-responsive nanomaterial for cancer chemo-photothermal therapy. RSC Adv..

[63] Tian B., Wang C., Zhang S., Feng L., Liu Z. (2011). Photothermally enhanced photodynamic therapy delivered by nano-graphene oxide. ACS Nano.

[64] Pandey S., Thakur M., Mewada A., Anjarlekar D., Mishra N., Sharon M. (2013). Carbon dots functionalized gold nanorod mediated delivery of doxorubicin: Tri-functional nano-worms for drug delivery, photothermal therapy and bioimaging. J. Mater. Chem. B.

[65] Zhou F., Xing D., Ou Z., Wu B., Resasco D.E., Chen W.R. (2009). Cancer photothermal therapy in the near-infrared region by using single-walled carbon nanotubes. J. Biomed. Opt..

[66] Sun Z., Xie H., Tang S., Yu X.F., Guo Z., Shao J., Zhang H., Huang H., Wang H., Chu P.K. (2015). Ultrasmall Black Phosphorus Quantum Dots: Synthesis and Use as Photothermal Agents. Angew. Chem. Int. Ed. Engl..

[67] Simelane N.W.N., Kruger C.A., Abrahamse H. (2021). Targeted Nanoparticle Photodynamic Diagnosis and Therapy of Colorectal Cancer. Int. J. Mol. Sci..

[68] Guan Q., Li Y.A., Li W.Y., Dong Y.B. (2018). Photodynamic Therapy Based on Nanoscale Metal-Organic Frameworks: From Material Design to Cancer Nanotherapeutics. Chem. Asian J..

[69] Velcheti V., Schalper K. (2016). Basic overview of current immunotherapy approaches in cancer. Am. Soc. Clin. Oncol. Educ. Book.

[70] Lakshmanan V.-K., Jindal S., Packirisamy G., Ojha S., Lian S., Kaushik A., Alzarooni A.I.M.A., Metwally Y.A.F., Thyagarajan S.P., Do Jung Y. (2021). Nanomedicine-based cancer immunotherapy: Recent trends and future perspectives. Cancer Gene Ther..

[71] Dogheim G.M., Nourhan E., Abd El-Maksod E.A., Amer S.S., El-Gizawy S.A., Abd Elhamid A.S., Elzoghby A.O. (2024). Nanomedicines as enhancers of tumor immunogenicity to augment cancer immunotherapy. Drug Discov. Today.

[72] Shi Y., Lammers T. (2019). Combining Nanomedicine and Immunotherapy. Acc. Chem. Res..

[73] Yang M., Li J., Gu P., Fan X. (2021). The application of nanoparticles in cancer immunotherapy: Targeting tumor microenvironment. Bioact. Mater..

[74] Chen Q., Feng L., Liu J., Zhu W., Dong Z., Wu Y., Liu Z. (2016). Intelligent albumin–MnO_2_ nanoparticles as pH-/H_2_O_2_-responsive dissociable nanocarriers to modulate tumor hypoxia for effective combination therapy. Adv. Mater..

[75] Basak U., Sarkar T., Mukherjee S., Chakraborty S., Dutta A., Dutta S., Nayak D., Kaushik S., Das T., Sa G. (2023). Tumor-associated macrophages: An effective player of the tumor microenvironment. Front. Immunol..

[76] Lee N.K., Kim S.-N., Park C.G. (2021). Immune cell targeting nanoparticles: A review. Biomater. Res..

[77] Ramishetti S., Kedmi R., Goldsmith M., Leonard F., Sprague A.G., Godin B., Gozin M., Cullis P.R., Dykxhoorn D.M., Peer D. (2015). Systemic Gene Silencing in Primary T Lymphocytes Using Targeted Lipid Nanoparticles. ACS Nano.

[78] Nawaz W., Xu S., Li Y., Huang B., Wu X., Wu Z. (2020). Nanotechnology and immunoengineering: How nanotechnology can boost CAR-T therapy. Acta Biomater..

[79] Siemaszko J., Marzec-Przyszlak A., Bogunia-Kubik K. (2021). NKG2D natural killer cell receptor—A short description and potential clinical applications. Cells.

[80] Thakur N., Thakur S., Chatterjee S., Das J., Sil P.C. (2020). Nanoparticles as smart carriers for enhanced cancer immunotherapy. Front. Chem..

[81] Chandrasekaran S., Chan M.F., Li J., King M.R. (2016). Super natural killer cells that target metastases in the tumor draining lymph nodes. Biomaterials.

[82] Gao S., Li T., Guo Y., Sun C., Xianyu B., Xu H. (2020). Selenium-containing nanoparticles combine the NK cells mediated immunotherapy with radiotherapy and chemotherapy. Adv. Mater..

[83] Mitarotonda R., Giorgi E., Eufrasio-da-Silva T., Dolatshahi-Pirouz A., Mishra Y.K., Khademhosseini A., Desimone M.F., De Marzi M., Orive G. (2022). Immunotherapeutic nanoparticles: From autoimmune disease control to the development of vaccines. Biomater. Adv..

[84] Murugan D., Murugesan V., Panchapakesan B., Rangasamy L. (2022). Nanoparticle Enhancement of Natural Killer (NK) Cell-Based Immunotherapy. Cancers.

[85] Tan L., Han S., Ding S., Xiao W., Ding Y., Qian L., Wang C., Gong W. (2017). Chitosan nanoparticle-based delivery of fused NKG2D–IL-21 gene suppresses colon cancer growth in mice. Int. J. Nanomed..

[86] Wu M.-R., James Cook W., Zhang T., Sentman C.L. (2014). Targeting multiple types of tumors using NKG2D-coated iron oxide nanoparticles. Nanotechnology.

[87] Melgoza-González E.A., Bustamante-Córdova L., Hernández J. (2023). Recent advances in antigen targeting to antigen-presenting cells in veterinary medicine. Front. Immunol..

[88] El-Sayed N., Korotchenko E., Scheiblhofer S., Weiss R., Schneider M. (2021). Functionalized multifunctional nanovaccine for targeting dendritic cells and modulation of immune response. Int. J. Pharm..

[89] Yao Y., Zhou Y., Liu L., Xu Y., Chen Q., Wang Y., Wu S., Deng Y., Zhang J., Shao A. (2020). Nanoparticle-based drug delivery in cancer therapy and its role in overcoming drug resistance. Front. Mol. Biosci..

[90] Kiaie S.H., Salehi-Shadkami H., Sanaei M.J., Azizi M., Shokrollahi Barough M., Nasr M.S., Sheibani M. (2023). Nano-immunotherapy: Overcoming delivery challenge of immune checkpoint therapy. J. Nanobiotechnology.

[91] Shan X., Gong X., Li J., Wen J., Li Y., Zhang Z. (2022). Current approaches of nanomedicines in the market and various stage of clinical translation. Acta Pharm. Sin. B.

[92] Yu M., Yang W., Yue W., Chen Y. (2022). Targeted Cancer Immunotherapy: Nanoformulation Engineering and Clinical Translation. Adv. Sci..

[93] Mundekkad D., Cho W.C. (2022). Nanoparticles in Clinical Translation for Cancer Therapy. Int. J. Mol. Sci..

[94] Franco R., Rivas-Santisteban R., Navarro G., Reyes-Resina I. (2021). Adenosine Receptor Antagonists to Combat Cancer and to Boost Anti-Cancer Chemotherapy and Immunotherapy. Cells.

[95] Miguel R.D.A., Hirata A.S., Jimenez P.C., Lopes L.B., Costa-Lotufo L.V. (2022). Beyond Formulation: Contributions of Nanotechnology for Translation of Anticancer Natural Products into New Drugs. Pharmaceutics.

[96] D’haese S., Lacroix C., Garcia F., Plana M., Ruta S., Vanham G., Verrier B., Aerts J.L. (2021). Off the beaten path: Novel mRNA-nanoformulations for therapeutic vaccination against HIV. J. Control. Release.

[97] Lin C.J., Lin Y.L., Luh F., Yen Y., Chen R.M. (2016). Preclinical effects of CRLX101, an investigational camptothecin-containing nanoparticle drug conjugate, on treating glioblastoma multiforme via apoptosis and antiangiogenesis. Oncotarget.

[98] Tada R., Hidaka A., Iwase N., Takahashi S., Yamakita Y., Iwata T., Muto S., Sato E., Takayama N., Honjo E. (2015). Intranasal Immunization with DOTAP Cationic Liposomes Combined with DC-Cholesterol Induces Potent Antigen-Specific Mucosal and Systemic Immune Responses in Mice. PLoS ONE.

[99] Doonan B., Shaw C., Lee J.-H., Manso E., Mendez-Gomez H., Roemeling C.V., Mitchell D.A., Sayour E. (2023). 772 Novel RNA-nanoparticle vaccine for the treatment of early melanoma recurrence following adjuvant anti-PD-1 antibody therapy. J. ImmunoTherapy Cancer.

[100] Borodovsky A., Barbon C.M., Wang Y., Ye M., Prickett L., Chandra D., Shaw J., Deng N., Sachsenmeier K., Clarke J.D. (2020). Small molecule AZD4635 inhibitor of A(2A)R signaling rescues immune cell function including CD103(+) dendritic cells enhancing anti-tumor immunity. J. Immunother. Cancer.

[101] Jia J., Zhang Y., Xin Y., Jiang C., Yan B., Zhai S. (2018). Interactions between nanoparticles and dendritic cells: From the perspective of cancer immunotherapy. Front. Oncol..

[102] Gonçalves G.A.R., Paiva R.M.A. (2017). Gene therapy: Advances, challenges and perspectives. Einstein (Sao Paulo).

[103] Wong C.H., Li D., Wang N., Gruber J., Lo A.W., Conti R.M. (2023). The estimated annual financial impact of gene therapy in the United States. Gene Ther..

[104] Scheller E.L., Krebsbach P.H. (2009). Gene therapy: Design and prospects for craniofacial regeneration. J. Dent. Res..

[105] Trafton A. Nanoparticles for Gene Therapy Improve. 6 November 2009. https://news.mit.edu/2009/nanoparticles-gene.

[106] Hamimed S., Jabberi M., Chatti A. (2022). Nanotechnology in drug and gene delivery. Naunyn-Schmiedeberg’s Arch. Pharmacol..

[107] Huang Y., Zhan Q., Wu C., Liao N., Jiang Z., Ding H., Wang K., Li Y. (2022). Trends and Hotspots in Nanoparticles for the Targeted Delivery of Nucleic Acids: A Ten-Year Bibliometric Study. Front. Pharmacol..

[108] Roma-Rodrigues C., Rivas-García L., Baptista P.V., Fernandes A.R. (2020). Gene Therapy in Cancer Treatment: Why Go Nano?. Pharmaceutics.

[109] Chen J., Guo Z., Tian H., Chen X. (2016). Production and clinical development of nanoparticles for gene delivery. Mol. Ther. Methods Clin. Dev..

[110] McBain S.C., Yiu H.H., Dobson J. (2008). Magnetic nanoparticles for gene and drug delivery. Int. J. Nanomed..

[111] Jin L., Wang Q., Chen J., Wang Z., Xin H., Zhang D. (2019). Efficient delivery of therapeutic siRNA by Fe_3_O_4_ magnetic nanoparticles into oral cancer cells. Pharmaceutics.

[112] Ryou S.-M., Kim J.-M., Yeom J.-H., Hyun S., Kim S., Han M.S., Kim S.W., Bae J., Rhee S., Lee K. (2011). Gold nanoparticle-assisted delivery of small, highly structured RNA into the nuclei of human cells. Biochem. Biophys. Res. Commun..

[113] Lee J.-M., Yoon T.-J., Cho Y.-S. (2013). Recent developments in nanoparticle-based siRNA delivery for cancer therapy. BioMed Res. Int..

[114] Taghavi S., Nia A.H., Abnous K., Ramezani M. (2017). Polyethylenimine-functionalized carbon nanotubes tagged with AS1411 aptamer for combination gene and drug delivery into human gastric cancer cells. Int. J. Pharm..

[115] Bhakta G., Sharma R.K., Gupta N., Cool S., Nurcombe V., Maitra A. (2011). Multifunctional silica nanoparticles with potentials of imaging and gene delivery. Nanomed. Nanotechnol. Biol. Med..

[116] Carvalho A.M., Cordeiro R.A., Faneca H. (2020). Silica-based gene delivery systems: From design to therapeutic applications. Pharmaceutics.

[117] Mirón-Barroso S., Domènech E.B., Trigueros S. (2021). Nanotechnology-based strategies to overcome current barriers in gene delivery. Int. J. Mol. Sci..

[118] Polack F.P., Thomas S.J., Kitchin N., Absalon J., Gurtman A., Lockhart S., Perez J.L., Pérez Marc G., Moreira E.D., Zerbini C. (2020). Safety and efficacy of the BNT162b2 mRNA COVID-19 vaccine. N. Engl. J. Med..

[119] Alvarez-Erviti L., Seow Y., Yin H., Betts C., Lakhal S., Wood M.J. (2011). Delivery of siRNA to the mouse brain by systemic injection of targeted exosomes. Nat. Biotechnol..

[120] Cheraghi R., Nazari M., Alipour M., Majidi A., Hosseinkhani S. (2016). Development of a targeted anti-HER2 scFv chimeric peptide for gene delivery into HER2-positive breast cancer cells. Int. J. Pharm..

[121] Jiang Z., Thayumanavan S. (2020). Noncationic material design for nucleic acid delivery. Adv. Ther..

[122] Choi Y.S., Lee M.Y., David A.E., Park Y.S. (2014). Nanoparticles for gene delivery: Therapeutic and toxic effects. Mol. Cell. Toxicol..

[123] Duan L., Ouyang K., Xu X., Xu L., Wen C., Zhou X., Qin Z., Xu Z., Sun W., Liang Y. (2021). Nanoparticle Delivery of CRISPR/Cas9 for Genome Editing. Front. Genet..

[124] Jürgens D.C., Deßloch L., Porras-Gonzalez D., Winkeljann J., Zielinski S., Munschauer M., Hörner A.L., Burgstaller G., Winkeljann B., Merkel O.M. (2023). Lab-scale siRNA and mRNA LNP manufacturing by various microfluidic mixing techniques—An evaluation of particle properties and efficiency. OpenNano.

[125] Zhu Y., Shen R., Vuong I., Reynolds R.A., Shears M.J., Yao Z.-C., Hu Y., Cho W.J., Kong J., Reddy S.K. (2022). Multi-step screening of DNA/lipid nanoparticles and co-delivery with siRNA to enhance and prolong gene expression. Nat. Commun..

[126] Khademi Z., Ramezani M., Alibolandi M., Zirak M.R., Salmasi Z., Abnous K., Taghdisi S.M. (2022). A novel dual-targeting delivery system for specific delivery of CRISPR/Cas9 using hyaluronic acid, chitosan and AS1411. Carbohydr. Polym..

[127] Casadidio C., Hartman J.E.M., Mesquita B.S., Haegebaert R., Remaut K., Neumann M., Hak J., Censi R., Di Martino P., Hennink W.E. (2023). Effect of Polyplex Size on Penetration into Tumor Spheroids. Mol. Pharm..

[128] Xue C., Hu S., Gao Z.-H., Wang L., Luo M.-X., Yu X., Li B.-F., Shen Z., Wu Z.-S. (2021). Programmably tiling rigidified DNA brick on gold nanoparticle as multi-functional shell for cancer-targeted delivery of siRNAs. Nat. Commun..

[129] Rohiwal S.S., Nguyen T.D., Kamenna E., Klima J., Vaskovicova M., Sekac D., Slouf M., Pavlova E., Stepanek P., Babuka D. (2023). Iron Oxide Nanoparticle-Mediated siRNA Delivery System for Huntington’s Disease Treatment. ACS Appl. Nano Mater..

[130] Liu C., Wan T., Wang H., Zhang S., Ping Y., Cheng Y. (2019). A boronic acid-rich dendrimer with robust and unprecedented efficiency for cytosolic protein delivery and CRISPR-Cas9 gene editing. Sci. Adv..

[131] Crivianu-Gaita V., Thompson M. (2016). Aptamers, antibody scFv, and antibody Fab’fragments: An overview and comparison of three of the most versatile biosensor biorecognition elements. Biosens. Bioelectron..

[132] Roacho-Perez J.A., Gallardo-Blanco H.L., Sanchez-Dominguez M., Garcia-Casillas P.E., Chapa-Gonzalez C., Sanchez-Dominguez C.N. (2018). Nanoparticles for death-induced gene therapy in cancer (Review). Mol. Med. Rep..

[133] Xu X., Liu C., Wang Y., Koivisto O., Zhou J., Shu Y., Zhang H. (2021). Nanotechnology-based delivery of CRISPR/Cas9 for cancer treatment. Adv. Drug Deliv. Rev..

[134] Li T., Yang Y., Qi H., Cui W., Zhang L., Fu X., He X., Liu M., Li P.-f., Yu T. (2023). CRISPR/Cas9 therapeutics: Progress and prospects. Signal Transduct. Target. Ther..

[135] Liu Q., Zhao K., Wang C., Zhang Z., Zheng C., Zhao Y., Zheng Y., Liu C., An Y., Shi L. (2019). Multistage delivery nanoparticle facilitates efficient CRISPR/dCas9 activation and tumor growth suppression in vivo. Adv. Sci..

[136] Li Z., Zhou X., Wei M., Gao X., Zhao L., Shi R., Sun W., Duan Y., Yang G., Yuan L. (2018). In vitro and in vivo RNA inhibition by CD9-HuR functionalized exosomes encapsulated with miRNA or CRISPR/dCas9. Nano Lett..

[137] Gessner I., Neundorf I. (2020). Nanoparticles Modified with Cell-Penetrating Peptides: Conjugation Mechanisms, Physicochemical Properties, and Application in Cancer Diagnosis and Therapy. Int. J. Mol. Sci..

[138] Ye S.-f., Tian M.-m., Wang T.-x., Ren L., Wang D., Shen L.-h., Shang T. (2012). Synergistic effects of cell-penetrating peptide Tat and fusogenic peptide HA2-enhanced cellular internalization and gene transduction of organosilica nanoparticles. Nanomed. Nanotechnol. Biol. Med..

[139] Taylor R.E., Zahid M. (2020). Cell Penetrating Peptides, Novel Vectors for Gene Therapy. Pharmaceutics.

[140] Huang S., Shao K., Liu Y., Kuang Y., Li J., An S., Guo Y., Ma H., Jiang C. (2013). Tumor-targeting and microenvironment-responsive smart nanoparticles for combination therapy of antiangiogenesis and apoptosis. ACS Nano.

[141] Davis M.E., Zuckerman J.E., Choi C.H.J., Seligson D., Tolcher A., Alabi C.A., Yen Y., Heidel J.D., Ribas A. (2010). Evidence of RNAi in humans from systemically administered siRNA via targeted nanoparticles. Nature.

[142] Ikada Y. (2006). Challenges in tissue engineering. J. R. Soc. Interface.

[143] Olson J.L., Atala A., Yoo J.J. (2011). Tissue engineering: Current strategies and future directions. Chonnam Med. J..

[144] Sudhakar C.K., Upadhyay N., Verma A., Jain A., Narayana Charyulu R., Jain S., Thomas S., Grohens Y., Ninan N. (2015). Chapter 1—Nanomedicine and Tissue Engineering. Nanotechnology Applications for Tissue Engineering.

[145] Fathi-Achachelouei M., Knopf-Marques H., Ribeiro da Silva C.E., Barthès J., Bat E., Tezcaner A., Vrana N.E. (2019). Use of Nanoparticles in Tissue Engineering and Regenerative Medicine. Front. Bioeng. Biotechnol..

[146] Shi J., Votruba A.R., Farokhzad O.C., Langer R. (2010). Nanotechnology in drug delivery and tissue engineering: From discovery to applications. Nano Lett..

[147] Jiang C., Wang K., Liu Y., Zhang C., Wang B. (2021). Using Wet Electrospun PCL/Gelatin/CNT Yarns to Fabricate Textile-Based Scaffolds for Vascular Tissue Engineering. ACS Biomater. Sci. Eng..

[148] Liu J., Wiley B., Chan I., Panchot A., Zong X., Cao Y., Offit K., Stadler Z., Link D., Bolton K. (2022). 43. Association between Clonal Hematopoiesis and Inherited Cancer Susceptibility Genes. Cancer Genet..

[149] Boguslavsky Y., Shemesh M., Friedlander A., Rutenberg R., Filossof A.M., Buslovich A., Poverenov E. (2018). Eliminating the Need for Biocidal Agents in Anti-Biofouling Polymers by Applying Grafted Nanosilica Instead. ACS Omega.

[150] Xi Y., Wang Y., Gao J., Xiao Y., Du J. (2019). Dual Corona Vesicles with Intrinsic Antibacterial and Enhanced Antibiotic Delivery Capabilities for Effective Treatment of Biofilm-Induced Periodontitis. ACS Nano.

[151] Aloma K.K., Sukaryo S., Fahlawati N.I., Dahlan K., Oemar S. (2020). Synthesis of Nanofibers from Alginate-Polyvinyl Alcohol using Electrospinning Methods. Macromol. Symp..

[152] He W., Ma Z., Yong T., Teo W.E., Ramakrishna S. (2005). Fabrication of collagen-coated biodegradable polymer nanofiber mesh and its potential for endothelial cells growth. Biomaterials.

[153] Ragothaman M., Villalan A.K., Dhanasekaran A., Palanisamy T. (2021). Bio-hybrid hydrogel comprising collagen-capped silver nanoparticles and melatonin for accelerated tissue regeneration in skin defects. Mater. Sci. Eng. C.

[154] Sang S., Cheng R., Cao Y., Yan Y., Shen Z., Zhao Y., Han Y. (2022). Biocompatible chitosan/polyethylene glycol/multi-walled carbon nanotube composite scaffolds for neural tissue engineering. J. Zhejiang Univ. Sci. B.

[155] Heidari M., Bahrami S.H., Ranjbar-Mohammadi M., Milan P.B. (2019). Smart electrospun nanofibers containing PCL/gelatin/graphene oxide for application in nerve tissue engineering. Mater. Sci. Eng. C.

[156] Li Y., Huang L., Tai G., Yan F., Cai L., Xin C., Al Islam S. (2022). Graphene Oxide-loaded magnetic nanoparticles within 3D hydrogel form High-performance scaffolds for bone regeneration and tumour treatment. Compos. Part A Appl. Sci. Manuf..

[157] Xie C., Satake-Ozawa M., Rashed F., Khan M., Ikeda M., Hayashi S., Sawada S., Sasaki Y., Ikeda T., Mori Y. (2022). Perforated Hydrogels Consisting of Cholesterol-Bearing Pullulan (CHP) Nanogels: A Newly Designed Scaffold for Bone Regeneration Induced by RANKL-Binding Peptides and BMP-2. Int. J. Mol. Sci..

[158] Borzenkov M., Chirico G., Collini M., Pallavicini P. (2018). Gold nanoparticles for tissue engineering. Environ. Nanotechnol..

[159] Mitchell M.J., Billingsley M.M., Haley R.M., Wechsler M.E., Peppas N.A., Langer R. (2021). Engineering precision nanoparticles for drug delivery. Nat. Rev. Drug Discov..

[160] Buyukhatipoglu K., Chang R., Sun W., Clyne A.M. Bioprinted nanoparticles for tissue engineering. Proceedings of the 2009 IEEE International Conference on Computational Intelligence for Measurement Systems and Applications.

[161] Wen X., Shi D., Zhang N. (2005). Applications of nanotechnology in tissue engineering. Handbook of Nanostructured Biomaterials and Their Applications in Nanobiotechnology.

[162] Bhutta Z.A., Kulyar M.F.-e.-A., Farooq U., Ashar A., Mahfooz A., Kanwal A., Akhtar M., Asif M., Nawaz S., Li K., Ul Islam S., Hussain C.M., Shukla S.K. (2023). Chapter 15—Applications of functionalized nanoparticles in tissue engineering. Antiviral and Antimicrobial Coatings Based on Functionalized Nanomaterials.

[163] Sharma K., Tharmatt A., Chawla P.A., Shah K., Chawla V., Sapra B., Bedi N. (2022). An Insight into Advanced Nanoparticles as Multifunctional Biomimetic Systems in Tissue Engineering. Nanopharmaceuticals in Regenerative Medicine.

[164] Zheng X., Zhang P., Fu Z., Meng S., Dai L., Yang H. (2021). Applications of nanomaterials in tissue engineering. RSC Adv..

[165] Yue Y., Luo H., Han J., Chen Y., Jiang J. (2020). Assessing the effects of cellulose-inorganic nanofillers on thermo/pH-dual responsive hydrogels. Appl. Surf. Sci..

[166] Yuan M., Wang Y., Qin Y.-X. (2018). Promoting neuroregeneration by applying dynamic magnetic fields to a novel nanomedicine: Superparamagnetic iron oxide (SPIO)-gold nanoparticles bounded with nerve growth factor (NGF). Nanomed. Nanotechnol. Biol. Med..

[167] Li Y., Guo Y., Niu W., Chen M., Xue Y., Ge J., Ma P.X., Lei B. (2018). Biodegradable multifunctional bioactive glass-based nanocomposite elastomers with controlled biomineralization activity, real-time bioimaging tracking, and decreased inflammatory response. ACS Appl. Mater. Interfaces.

[168] Villarreal Gómez L.J. (2022). Electrospun Nanofibers for Tissue Engineering: Desired Properties. Open Biomater. Sci. J..

[169] Alizadeh P., Soltani M., Tutar R., Hoque Apu E., Maduka C.V., Unluturk B.D., Contag C.H., Ashammakhi N. (2021). Use of electroconductive biomaterials for engineering tissues by 3D printing and 3D bioprinting. Essays Biochem..

[170] Ziv-Polat O., Margel S., Shahar A. (2015). Application of iron oxide anoparticles in neuronal tissue engineering. Neural Regen. Res..

[171] Hasan A., Morshed M., Memic A., Hassan S., Webster T.J., Marei H.E. (2018). Nanoparticles in tissue engineering: Applications, challenges and prospects. Int. J. Nanomed..

[172] Arulpriya P., Krishnaveni T., Lakshmi K., Kadirvelu K. (2022). Bionanomaterials and Their Recent Advancements in Tissue Engineering Applications. Nanophytomedicine.

[173] Loukelis K., Helal Z.A., Mikos A.G., Chatzinikolaidou M. (2023). Nanocomposite Bioprinting for Tissue Engineering Applications. Gels.

[174] Zhang K., Wang S., Zhou C., Cheng L., Gao X., Xie X., Sun J., Wang H., Weir M.D., Reynolds M.A. (2018). Advanced smart biomaterials and constructs for hard tissue engineering and regeneration. Bone Res..

[175] Plotkin S. (2014). History of vaccination. Proc. Natl. Acad. Sci. USA.

[176] Bezbaruah R., Chavda V.P., Nongrang L., Alom S., Deka K., Kalita T., Ali F., Bhattacharjee B., Vora L. (2022). Nanoparticle-based delivery systems for vaccines. Vaccines.

[177] Peiffer-Smadja N., Rozencwajg S., Kherabi Y., Yazdanpanah Y., Montravers P. (2021). COVID-19 vaccines: A race against time. Anaesth. Crit. Care Pain Med..

[178] Ramirez J.E.V., Sharpe L.A., Peppas N.A. (2017). Current state and challenges in developing oral vaccines. Adv. Drug Deliv. Rev..

[179] Almotairy A., Yusuf A., Henidi H., Alshehri O., Aldughaim M. (2023). Nanoparticles as Drug Delivery Systems: A Review of the Implication of Nanoparticles’ Physicochemical Properties on Responses in Biological Systems. Polymers.

[180] Pascolo S. (2021). Synthetic messenger RNA-based vaccines: From scorn to hype. Viruses.

[181] Arevalo C.P., Bolton M.J., Le Sage V., Ye N., Furey C., Muramatsu H., Alameh M.-G., Pardi N., Drapeau E.M., Parkhouse K. (2022). A multivalent nucleoside-modified mRNA vaccine against all known influenza virus subtypes. Science.

[182] Marrack P., McKee A.S., Munks M.W. (2009). Towards an understanding of the adjuvant action of aluminium. Nat. Rev. Immunol..

[183] Kim H., Niu L., Larson P., Kucaba T.A., Murphy K.A., James B.R., Ferguson D.M., Griffith T.S., Panyam J. (2018). Polymeric nanoparticles encapsulating novel TLR7/8 agonists as immunostimulatory adjuvants for enhanced cancer immunotherapy. Biomaterials.

[184] Stickdorn J., Stein L., Arnold-Schild D., Hahlbrock J., Medina-Montano C., Bartneck J., Ziß T., Montermann E., Kappel C., Hobernik D. (2022). Systemically administered TLR7/8 agonist and antigen-conjugated nanogels govern immune responses against tumors. ACS Nano.

[185] Lee J.H., Chapman D.V., Saltzman W.M. (2023). Nanoparticle targeting with antibodies in the central nervous system. BME Front..

[186] Park K.S., Bazzill J.D., Son S., Nam J., Shin S.W., Ochyl L.J., Stuckey J.A., Meagher J.L., Chang L., Song J. (2021). Lipid-based vaccine nanoparticles for induction of humoral immune responses against HIV-1 and SARS-CoV-2. J. Control. Release.

[187] Sia Z.R., He X., Zhang A., Ang J.C., Shao S., Seffouh A., Huang W.-C., D’Agostino M.R., Teimouri Dereshgi A., Suryaprakash S. (2021). A liposome-displayed hemagglutinin vaccine platform protects mice and ferrets from heterologous influenza virus challenge. Proc. Natl. Acad. Sci. USA.

[188] Hou X., Zaks T., Langer R., Dong Y. (2021). Lipid nanoparticles for mRNA delivery. Nat. Rev. Mater..

[189] Linkov I., Satterstrom F.K., Corey L.M. (2008). Nanotoxicology and nanomedicine: Making hard decisions. Nanomed. Nanotechnol. Biol. Med..

[190] Miller M.R., Poland C.A. (2020). Nanotoxicology: The need for a human touch?. Small.

[191] Feliu N., Fadeel B. (2010). Nanotoxicology: No small matter. Nanoscale.

[192] Forest V. (2022). Experimental and computational nanotoxicology—Complementary approaches for nanomaterial hazard assessment. Nanomaterials.

[193] Domingues C., Santos A., Alvarez-Lorenzo C., Concheiro A., Jarak I., Veiga F., Barbosa I., Dourado M., Figueiras A. (2022). Where is nano today and where is it headed? A review of nanomedicine and the dilemma of nanotoxicology. ACS Nano.

